# Inter-hospital and inter-disciplinary variation in planned birth practices and readiness for change: a survey study

**DOI:** 10.1186/s12884-021-03844-z

**Published:** 2021-05-20

**Authors:** Dominiek Coates, Natasha Donnolley, Maralyn Foureur, Amanda Henry

**Affiliations:** 1 Faculty of Health, Centre for Midwifery and Child and Family Health, University of Technology Sydney, Sydney, Australia; 2 Maridulu Budyari Gumal, the Sydney Partnership for Health, Education, Research and Enterprise (SPHERE), Sydney, Australia; 3 National Perinatal Epidemiology and Statistics Unit, Centre for Big Data Research in Health, UNSW, Sydney, Australia; 4 Hunter New England Nursing and Midwifery Research Centre, Newcastle, Australia; 5 Faculty of Health and Medicine, University of Newcastle, Newcastle, Australia; 6 School of Women’s and Children’s Health, UNSW Medicine, UNSW, Sydney, Australia; 7 Department of Women’s and Children’s Health, St George Hospital, Sydney, Australia; 8 The George Institute for Global Health, UNSW Medicine, Sydney, Australia

**Keywords:** Caesarean section, Induction of labour, Unwarranted clinical variation, Practice change, Shared decision-making

## Abstract

**Background:**

How the application of evidence to planned birth practices, induction of labour (IOL) and prelabour caesarean (CS), differs between Australian maternity units remains poorly understood. Perceptions of readiness for practice change and resources to implement change in individual units are also unclear.

**Aim:**

To identify inter-hospital and inter-professional variations in relation to current planned birth practices and readiness for change, reported by clinicians in 7 maternity units.

**Method:**

Custom-created survey of maternity staff at 7 Sydney hospitals, with questions about women’s engagement with decision making, indications for planned birth, timing of birth and readiness for change. Responses from midwives and medical staff, and from each hospital, were compared.

**Findings:**

Of 245 completed surveys (27% response rate), 78% were midwives and 22% medical staff. Substantial inter-hospital variation was noted for stated planned birth indication, timing, women’s involvement in decision-making practices, as well as in staff perceptions of their unit’s readiness for change. Overall, 48% (range 31–64%) and 64% (range 39–89%) agreed on a need to change their unit’s caesarean and induction practices respectively. The three units where greatest need for change was perceived also had least readiness for change in terms of leadership, culture, and resources. Regarding inter-disciplinary variation, medical staff were more likely than midwifery staff to believe women were appropriately informed and less likely to believe unit practice change was required.

**Conclusion:**

Planned birth practices and change readiness varied between participating hospitals and professional groups. Hospitals with greatest perceived need for change perceived least resources to implement such change.

**Supplementary Information:**

The online version contains supplementary material available at 10.1186/s12884-021-03844-z.

## Introduction

While it is increasingly recognised that evidence is not routinely implemented into practice [1, 2], it is less clear which evidence-based practices have been adopted and which are specified in policies and guidelines but not translated into everyday care [3, 4]. Inconsistent uptake of evidence has resulted in unwarranted variation in practices, across all areas of healthcare [3, 4], including maternity care [2]. Within maternity care, practice variation in relation to planned birth, i.e. induction of labour (IOL) and prelabour caesarean section (CS), is of increasing concern [2].

There has been a worldwide rise in IOL and CS over recent decades, with one in four births by CS [5, 6] and one in three by IOL [5, 7, 8] in many high-income countries. While, when medically indicated, planned birth can be lifesaving, unnecessary planned birth can lead to short-term or long-term adverse health impacts on women and children, particularly for CS [9, 10]. The extent to which planned birth is warranted remains unclear, with widespread variation in the incidence of planned birth between countries and hospitals [10–16]. For example, in New South Wales (NSW), Australia, IOL rates range from 9.7 to 41.2% [15] and CS rates range from 11.8 to 47.4% [12].

While evidence-based guidelines with recommendations regarding the decision of when to induce labour or perform a CS exist [17, 18], the extent to which these recommendations are actually followed remains unclear [2, 19]. Even though analyses of routinely collected patient data indicate that variation in planned birth practices exist [12, 15], these data are limited by the nature and quality of the data collected. While hospital data can tell us about the number of IOL and CS performed, and the indications for IOL and CS, it does not tell us about the specific hospital practices. Similarly, while evidence from qualitative and survey studies indicate that planned birth decision-making is informed by clinician preferences and the culture of the unit (influenced by a wide range of factors including hierarchical structure, interprofessional trust, philosophy of care, risk guidelines and management policies) [20–22], these studies provide little insight into the specific practices, i.e. what occurs in practice, and how this varies between units. We have previously reported, for example, on inter- and intra-professional midwifery and medical variation in which planned birth indications are considered “valid” in our study hospitals [23], however how this translates to actual planned birth practices in the relevant units remains unknown.

As well as a lack of understanding of how planned birth practices differ between Australian maternity units, there is also a lack of knowledge around unit readiness to change their practices and reduce unwarranted variation, and this is the omission this study sought to address. Organisational readiness for change in healthcare settings refers to ‘the extent to which organisational members are psychologically and behaviourally prepared to implement organisational change’ [24], and ascertaining readiness for change is important to successfully implement new policies, programs, and practices [25, 26]. When readiness for change is high, members are more likely to initiate change, exert greater effort, and display more cooperative behaviour, resulting in more effective implementation of change [27].

The aim of this study was therefore to identify inter-hospital and inter-professional variations in relation to current planned birth practices, and readiness for change, reported by clinicians in 7 maternity units. Specifically, we aimed to (a) compare maternity unit practices amongst the participating hospitals in relation to indications for and timing of planned birth, including decision-making practices (b) assess perceptions around the need for, and readiness for change, as reported by clinicians in the participating maternity units, and (c) to compare responses inter-professionally (midwifery versus medical) regarding current planned birth practices and need/readiness for change. Given the ultimate aim of the overarching study (Timing of Birth) was to inform practice change, we were interested in examining both planned birth practices as well as perception of the need for and readiness for change.

## Method

A survey study was conducted to identify planned birth practices and readiness for change of 7 public maternity hospitals (8 units, representing all the public maternity units in the two included regions of Sydney, were invited to participate but 1 was excluded due to low response rate/low clinician numbers). The participating public hospitals were chosen as they were actively engaged in a clinical research partnership with the researchers via an umbrella organisation, Sydney Partnership for Health, Education, *Research* and Enterprise (SPHERE). The 7 hospitals are from two health districts, cover approximately 20% of the total births in NSW (of 69 public maternity hospitals in NSW) and encompass socio-demographically diverse communities representative of the broader Australian multicultural maternity population [5], and include units with a range of service capabilities, from those offering only low-risk term (37 + weeks) intrapartum care (Level 3), to 34 weeks and above care (Level 4), planned 32 weeks and above care with accompanying special care nursery capabilities (Level 5), and care of the most complex pregnancies at any gestation with accompanying full neonatal intensive care facilities (Level 6) [28].

All included units provide antenatal care in line with their intrapartum service capabilities (and transfer care to a higher level facility in the region if complications develop), and encompass a variety of models of pregnancy and birth care (e.g. caseload midwifery-led, and medically-led). As is standard for Australian public maternity units, midwives are the primary accoucheurs for the majority of births, with medical staff involved if intervention is required.

All methods were carried out in accordance with the Declaration of Helsinki and the Australian Code for the Responsible Conduct of Research (2018) [29]. Informed consent was received from all participants, with ethics approval received from the Eastern Sydney Local Health Human Research Ethics Committee (18/169 HREC/18/POWH/356).

### The survey

A custom-created survey was developed by the authors, whose backgrounds include a social scientist, midwifery clinician and academic, obstetric clinician and academic, and consumer representative. The survey development was informed by a review of existing guidelines [17, 18], as well as extensive feedback from maternity clinicians around which issues or practices warrant investigation. Relevant guidelines existing at the participating hospitals were examined, which confirmed to the research team that some degree of inter-hospital variation would be expected based on differing local protocols alone.

The face validity of the survey was tested through the pilot process. The survey was tested by 10 maternity clinicians (midwifery and medical) known to the research team, who provided feedback which informed amendments. The survey consisted of a range of multiple-choice lists and Likert-scaled questions (the specific statements are listed in the Tables as well as supplementary file 1). Multiple-choice lists were used to capture demographic information such as respondent discipline, primary area of care provision (antenatal, postnatal, intrapartum, management, etc.), age, sex, years of experience and affiliated hospital.

A 5-item Likert scale, from ‘strongly disagree’ to ‘strongly agree’ (with the middle option termed ‘undecided’), was used to capture practices in relation to women’s engagement with decision-making (4 items), indications for IOL and planned CS (18 items), and the timing of CS in participating units. Respondents also rated 19 multiple choice questions about the timing of IOL (in weeks gestation). Respondents were asked to choose the single most correct option, assuming for each indication that it is the only reason for IOL, and that the woman does not have a contraindication to vaginal birth. There was also a “not a reason for IOL” option in the timing questions, which was explained to participants “does not mean women in your unit are *never* induced for this reason: it indicates that it is not standard practice to induce for this reason/IOL for this reason would only be on a case by case basis”.

To capture readiness for change, respondents rated 14 Likert scale items about their unit’s need for changes to planned birth practices, and change readiness. Readiness for change questions included selected questions from a self-improvement toolkit that focuses on normal birth and reducing CS developed by the NHS Institute for Innovation and Improvement [30] as well as selected questions from Helfrich et al.’s (2009) Organizational Readiness to Change Assessment (ORCA) – Context scale [25]. The ORCA instrument consists of three major scales that measure 1) strength of the evidence for the proposed change/innovation; 2) quality of the organisational context to support the practice change; and 3) organisational capacity to facilitate the change.

### Participants and recruitment

All midwives (*N* = 750) and obstetric medical staff (*N* = 150) affiliated with the participating sites were eligible to participate. The survey was available electronically and in hard copy, with staff emailed the survey up to three times between November 2018 and July 2019. A senior member of staff at each of the participating sites (leader/manager/director) was asked to distribute the anonymous survey (via electronic link), as well as make paper copies of the survey available. Paper copies were distributed with reply-paid envelopes so completed surveys could be returned confidentially to the research team. As recruitment was managed by the participating hospitals, we are unsure exactly how many clinicians were invited to complete the survey, but believe all clinicians were informed of the study at least once. Respondents were informed that participation was voluntary and anonymous. (The same method was used in our study on clinician attitudes in relation to planned birth practices [23]).

### Data analysis

All survey responses were entered into REDCap, a customisable web-based research data collection and administration application and exported into Excel v16.27 and IBM SPSS Statistics 23 for analysis. Demographical data and Likert Scale responses were analysed using descriptive statistics. Responses are presented as whole numbers and percentages. For Likert scale responses, the percentages reflect level of agreement (% of respondents who agreed or strongly agreed with the statement).

Inferential statistics were used to compare Likert Scale responses. To test for differences in Likert scale responses (ordinal data) between respondents based on discipline, a Mann-Whitney u test was used because the data was not normally distributed on Shapiro-Wilk testing. To test for differences in responses between hospitals, Kruskal-Wallis test was used (3 or more independent groups). Significance was set at *p* < 0.05.

## Results

After removal of missing data, 245 responses were included, comprising 191 (78%) midwives and 54 (22%) medical staff (responses with more than 5% missing data were excluded). This constitutes an estimated response rate of 27% overall, 25% for midwives and 33% for medical staff (see Table 1). More midwives were female than medical staff (98% versus 68%), just under half of midwives and medical staff were 40 and under (43 and 44% respectively), and around 40% of both midwives and medical staff had over 15 years of experience (40 and 43% respectively).

**Table 1:** Clinician demographical data and hospital response rate (*N* = 245)

Value	Midwives (***N*** = 191) n (%)	Medical staff (***N*** = 54) n (%)
**Gender**		
Female	188 (98)	36 (68)
Male	1 (1)	15 (28)
Not stated	2 (1)	3 (4)
**Age, yrs**		
20–30	43 (23)	7 (13)
31–40	40 (21)	17 (31)
41–50	46 (24)	16 (30)
51–60	46 (24)	9 (17)
Over 60	13 (2)	5 (9)
Prefer not to say	3 (2)	–
**Years of experience**		
≥ 16 or more	79 (41)	24 (44)
Between 11 and 15	38 (20)	6 (11)
Between 5 and 10	37 (19)	12 (22)
< 5	36 (19)	11 (20)
Prefer not to say	1 (1)	1 (2)
**Midwives primary area**		
All areas	25 (13)	–
Antenatal care	34 (18)	–
Clinical midwifery consultant, specialist or educator	36 (19)	–
Intrapartum care	56 (29)	–
Management	8 (4)	–
Midwifery Group Practice	11 (6)	–
Postnatal care	17 (9)	–
Blank	4 (2)	–
**Obstetric medical staff primary area**		
Obstetric Registrar/Resident	–	22 (41)
Obstetrician, work predominantly public	–	15 (28)
Obstetrician, work equal public and private	–	12 (22)
Obstetrician, work predominantly private	–	5 (9)
**Participating hospitals, level**^a^		
Hospital A, level 6 (*n* = 78, 32%)	57 (73)	21 (27)
Hospital B, level 6 (*n* = 36, 15%)	31 (86)	5 (14)
Hospital C, level 5 (*n* = 37, 15%)	25 (68)	12 (32)
Hospital D, level 4 (*n* = 18, 7%)	14 (78)	4 (22)
Hospital E, level 4 (*n* = 36, 15%)	30 (84)	6 (16)
Hospital F, level 4 (*n* = 20, 8%)	17 (88)	3 (12)
Hospital G, level 3 (*n* = 20, 8%)	17 (86)	3 (14)

Level 3: Provides planned intrapartum care for women ≥37 + 0 weeks gestation and immediate care for birth≥34 weeks – if any complications transfer to higher level [4–6] neonatal care

Level 4: Provides planned intrapartum care for women ≥34 + 0 weeks gestation

Level 5: Provides planned intrapartum care for women ≥32 weeks, and level 4 neonatal service (special care nursery with ability for ongoing respiratory support with high flow oxygen and continuous positive aware pressure, and capacity for emergency intubation and mechanical ventilation prior to transfer to Level 5 or 6 neonatal service)

Level 6: Provides planned intrapartum care for women of any gestation or obstetric risk, has level 5 or 6 neonatal service (full neonatal intensive care services)

^a^ Hospital levels as per the NSW Maternity and Neonatal Service Capability [28]

### Planned birth practices: inter-hospital variation

As indicated in Table 2, there was statistically significant inter-hospital variation in women’s involvement in decision-making, for all 4 Likert scale items. While overall 73% of clinicians agreed or strongly agreed that women are informed about the benefits and risks of interventions such as IOL and CS, this ranged from 22 to 95% between hospitals. While 59% agreed or strongly agreed that women are supported to make decisions about their own care in relation to IOL, this ranged from 25 to 79%. Similarly, while 56% of clinicians agreed that women are supported to make decisions about their own care in relation to CS, this ranged from 22 to 78%. While 67% agreed or strongly agreed that consideration is given to the wishes and preferences of the woman in decisions about her care in relation to IOL and CS this ranged from 36 to 84%.

**Table 2:** Practices in relation to decision-making and indications for planned birth - % of respondents who agreed or strongly agreed with the statement

	% of all respondents	Hospital A, level 6 ***n*** = 78	Hospital B, level 6 ***n*** = 36	Hospital C, level 5 ***n*** = 37	Hospital D, level 4 ***n*** = 18	Hospital E, level 4 ***n*** = 36	Hospital F, level 4 ***n*** = 20	Hospital G, level 3 ***n*** = 20	***p***-value	Midwives ***n*** = 191	Medical staff ***n*** = 54	***p***-value
**Women’s involvement in decision-making**												
Women are informed about the benefits and risks of interventions such as IOL and CS	73	90	69	73	84	22	95	75	< 0.001*	68	93	.007*
Women are supported to make decisions about their own care in relation to IOL	59	79	45	59	67	25	60	70	< 0.001*	51	89	< 0.001*
Women are supported to make decisions about their own care in relation to CS	56^a^	64	38	78	62	22	70	60	< 0.001*	49	81	< 0.001*
Consideration is given to the wishes and preferences of the woman in decisions about her care in relation to IOL and CS	67	84	49	77	73	36	80	70	< 0.001*	61	91	< 0.001*
**Indications for planned birth**												
In terms of the management of prolonged pregnancy, women from some ethnic groups, such as South Asian women, are induced at earlier gestations	34	15	23	97	28	14	90	10	< 0.001*	31	45	.110
Women with uncomplicated DCDA twin pregnancies, where the presenting twin is cephalic, are supported to have a vaginal birth	75	87	80	89	95	66	50	35	< 0.001*	71	89	.011*
Women with uncomplicated MCDA twin pregnancies, where the presenting twin is cephalic, are supported to have a vaginal birth	55^a^	67	61	64	78	43	25	30	< 0.001*	50	73	.003*
Women who are afraid of childbirth are counselled and provided with information about the pros and cons of CS	68	78	67	65	78	55	65	60	.218	64	84	.004*
Women who request a CS (without a medical reason) are counselled and supported to have a vaginal birth	73	75	72	79	67	69	90	55	.471	71	82	.182
Women who had a previous CS are supported to have a vaginal birth	82	88	78	97	100	72	65	90	< 0.001*	89	93	.073
Women with an uncomplicated breech (frank or complete breech, normal fetal size and welfare) are supported to have a vaginal birth, either in our unit or by referral to a unit which offers vaginal breech birth	50	90	25	51	28	14	35	35	< 0.001*	45	65	.019*
Women who had a previous uterine rupture are routinely recommended a CS	82	88	72	92	89	64	75	85	.103	78	96	.001*
Women with abnormal fetal lie (e.g. transverse lie), where ECV is declined, unsuccessful or not appropriate, are routinely recommended a CS	92	95	86	95	89	94	90	95	.477	91	98	.274
Women with abnormal fetal lie (e.g. breech or transverse) are routinely offered a CS in preference to ECV	24	15	28	19	33	42	25	50	< 0.001*	28	22	< 0.001*
Women with prior classical or inverted T uterine incision are recommended a repeat CS	90	95	86	100	77	86	80	80	.386	87	98	.033*
Women with previous perineal trauma (e.g. 3rd or 4th degree tear/obstetric anal sphincter injury) are offered a CS	75	61	86	76	61	86	85	90	.037*	75	78	.913
Women with previous severe pelvic floor damage (e.g. prolapse) are offered a CS	67^a^	62	72	76	50	80	75	75	.071	69	56	.097
Women with a previous fetal death in utero are routinely offered a CS	12^a^	13	8	8	23	8	20	5	.579	12	9	.089
Women with previous shoulder dystocia are routinely offered a CS	20^a^	5	34	27	11	25	25	40	.039*	19	27	.311
Women who are colonised with Group B Strep are offered a CS	1	1	3	0	0	0	0	0	.486	1	0	.070
In our unit, women with uncomplicated pregnancies are offered IOL before 41 + 0 weeks	14	5	31	8	11	25	5	25	.002*	18	2	.003*
In our unit, planned CS for singleton pregnancy where there is no maternal or fetal indication for early birth, are offered before 39 + 0 weeks	18	6	34	10	11	37	5	26	.011*	11	6	.281

*ECV* External cephalic version, *CS* Caesarean section, *DCDA* Dichorionic diamniotic, *MCDA* Monochorionic diamniotic

*statistical significance at < 0.05

^a^% of respondents who were undecided > 20% (range: 21 to 30%)

In relation to indications for planned birth, as indicted in Table 2, there was statistically significant inter-hospital variation for 9 out of the 18 items. Those with the greatest variation in stated practice included whether South Asian origin mothers undergo IOL earlier for prolonged pregnancy (range 10–97%), support for uncomplicated dichorionic, diamniotic (DCDA) or monochorionic, diamniotic (MCDA) twins to have a vaginal birth (range 35–95% and 25–78% respectively), support for uncomplicated vaginal breech birth, either in the unit or by referral on (14–90%), and support for vaginal birth after CS (range 65–100%). There was also inter-hospital variation in stated planned birth timing practices, with the range of planned CS < 39 weeks (no indication for earlier birth) being 5–37% and IOL of uncomplicated pregnancies before 41 + 0 weeks ranging from 5 to 31%.

Some of these variations reflect, at least in part, the different hospital levels (as described in Table 1), with lower level hospitals less likely to provide intrapartum care for twin pregnancies, breech pregnancies, and previous shoulder dystocia (due to restrictions on their service capability). However, in relation to both breech pregnancies, and previous shoulder dystocia this variation is not entirely consistent with varying hospital level: for example, only 25% of clinicians at a level 6 hospital (hospital B) agreed that women with an uncomplicated breech are supported to have a vaginal birth.

In relation to the timing of IOL, as shown in Table 3, there are a multitude of conditions where some clinicians believe it is advised to routinely induce before 39 weeks. An average of 6% (with a range from 0 to 18%) of respondents agreed that in their unit women are induced without a medical reason from 38 weeks. There was also considerable inter-hospital variation in relation to the timing of IOL for several indications, particularly diabetes, preeclampsia, maternal age of 40 and over, and elevated BMI (> 40 kg/m2) (note supplementary file 2 for figures of the data included in Table 3). Regarding diabetes, greatest variation was seen regarding insulin controlled gestational diabetes, with units split on IOL at 38+ versus 39+ weeks, and pre-existing diabetes (both Type I and Type II), with units split across IOL at 37+, 38+ and 39+ weeks. Regarding preeclampsia, there was also lack of consensus on when this was an indication for IOL, with on average 29, 29, 26 and 11% stating their unit offered IOL at 37+, 38+, 39+ and not until 40+ weeks respectively. For maternal age of 40+ and BMI of 40+, the main variation was between offering IOL at 39+ versus 40+ weeks (range 23–63% and 18–62% respectively for maternal age, and 13–38% and 19–59% respectively for BMI of 40+), however there was also variation for high BMI in particular as to whether this was a routine IOL indication (“not an indication” average 15%, range 3–44%). Variation in timing of twin IOL was also seen, in line with overall variation in twin planned birth practices noted in Table 2.

**Table 3:** Survey respondents’ perceptions of practices in relation to the timing of IOL – average and range (min-max) of responses between units

		35 + 0 weeks onwards	36 + 0 weeks onwards	37 + 0 weeks onwards	38 + 0 weeks onwards	39 + 0 weeks onwards	40 + 0 week onwards	41 + 0 weeks onwards	Not a reason for IOL^a^
Suspected macrosomia	**Average**	0%	1%	8%	30%	36%	13%	1%	11%
	**Range**	0–0%	0–10%	0–25%	13–44%	21–66%	0–25%	0–2%	0–25%
Request for IOL without medical indications	**Average**	0%	0%	0%	6%	19%	19%	18%	38%
	**Range**	0–0%	0–3%	0–0%	0–18%	3–32%	6–38%	0–23%	14–52%
Gestational diabetes that is diet controlled	**Average**	0%	0%	1%	10%	19%	37%	19%	13%
	**Range**	0–0%	0–3%	0–7%	0–26%	10–38%	32–69%	0–45%	0–23%
Gestational diabetes managed with oral hypoglycaemics (e.g. metformin)	**Average**	0%	0%	1%	22%	36%	33%	3%	5%
	**Range**	0–0%	0–3%	0–7%	3–46%	29–56%	18–55%	0–7%	0–5%
Insulin-requiring gestational diabetes (not pre-pregnancy Type I or II)	**Average**	0%	0%	6%	41%	41%	9%	0%	2%
	**Range**	0–3%	0–0%	0–25%	15–61%	11–62%	0–23%	0–0%	0–3%
Pre-pregnancy diabetes, Type I	**Average**	0%	0%	17%	47%	26%	8%	0%	2%
	**Range**	0–3%	0–0%	0–46%	23–63%	7–46%	0–15%	0–0%	0–8%
Pre-pregnancy diabetes, Type II	**Average**	0%	0%	8%	47%	31%	11%	0%	3%
	**Range**	0–3%	0–0%	0–25%	23–68%	7–48%	0–25%	0–2%	0–8%
Gestational hypertension (new-onset high blood pressure after 20 weeks, no preeclampsia)	**Average**	0%	0%	14%	33%	30%	16%	2%	5%
	**Range**	0–0%	0–3%	5–29%	23–44%	21–38%	4–23%	0–4%	0–0%
Chronic/essential hypertension	**Average**	0%	0%	14%	35%	27%	16%	2%	6%
	**Range**	0–0%	0–3%	5–29%	23–50%	16–38%	4–24%	0–3%	0–8%
Preeclampsia (assume no urgent indication for birth)	**Average**	0%	0%	29%	29%	26%	11%	2%	3%
	**Range**	0–4%	0–0%	0–59%	14–46%	11–50%	0–23%	0–5%	0–6%
Uncomplicated monochorionic diamniotic (MCDA) twin pregnancies (if vaginal birth is planned)	**Average**	2%	27%	30%	19%	8%	3%	0%	11%
	**Range**	0–13%	6–52%	13–45%	0–32%	4–19%	0–15%	0–0%	0–44%
Uncomplicated dichorionic diamniotic (DCDA) twin pregnancies (if vaginal birth is planned)	**Average**	0%	5%	29%	37%	12%	6%	0%	10%
	**Range**	0–3%	0–11%	6–59%	15–50%	3–31%	0–15%	0–0%	0–44%
Cholestasis of pregnancy	**Average**	1%	5%	55%	21%	10%	4%	1%	3%
	**Range**	0–6%	0–21%	32–79%	6–32%	0–25%	0–15%	0–4%	0–6%
Maternal age of 40 and over	**Average**	0%	0%	5%	11%	37%	43%	0%	5%
	**Range**	0–0%	0–0%	0–16%	6–25%	23–63%	18–62%	0–0%	0–16%
Substantially elevated BMI (> 40 kg/m2)	**Average**	0%	1%	3%	13%	25%	41%	2%	15%
	**Range**	0–0%	0–6%	0–13%	0–29%	13–38%	19–59%	0–7%	3–44%

^a^the “not a reason for IOL” option does not mean women in your unit are *never* induced for this reason: it indicates that it is not standard practice to induce for this reason/IOL for this reason would only be on a case by case basis

The other substantive variation in the use and timing of IOL relates to prelabour rupture of membranes (PROM), both term and preterm (Table 4). Even for term (37+ weeks) PROM, an average of 14% (range 6–26%) and 16% (range 6–25%) of respondents stated that women with Group B Strep (GBS)-positive and GBS-negative term PROM respectively were not offered IOL for this indication in their unit. Although most women with GBS-positive term PROM were offered IOL as soon as possible, within 12 h, or within 24 h (average 44, 15 and 14% respectively), only 35% of women who were GBS-negative were offered IOL after term PROM within 24 h.

**Table 4:** Practices in relation to the timing of IOL for *prelabour rupture of membranes (PROM) women with no other complications or signs of chorioamnionitis:* Average and range (min-max) between units

		ASAP	within 12 h	13–24 h	25–48 h	49–96 h	After 96 h	At 37 + 0 weeks	At 38 + 0 weeks	At 39 + 0 weeks	At 40 + 0 weeks	Not induced
A**t term** (37 + 0) who are Group B streptococcus positive	**Average**	44%	15%	14%	9%	0%	0%	2%	1%	0%	0%	14%
	**Range**	16–86%	7–29%	0–29%	0–19%	0–0%	0–0%	0–8%	0–10%	0–0%	0–6%	6–26%
A**t term** (> 37 + 0) who are Group B streptococcus negative	**Average**	2%	3%	30%	39%	6%	1%	1%	2%	0%	0%	16%
	**Range**	0–7%	0–7%	6–46%	23–81%	0–10%	0–3%	0–6%	0–10%	0–0%	0–6%	6–25%
**Late preterm** (34 + 0–36 + 6 weeks) who are Group B streptococcus positive	**Average**	30%	11%	8%	4%	2%	1%	23%	1%	0%	0%	19%
	**Range**	19–45%	0–28%	0–19%	0–13%	0–8%	0–6%	0–38%	0–6%	0–3%	0–6%	0–44%
**Late preterm** (34 + 0–36 + 6 weeks) who are Group B streptococcus negative	**Average**	3%	3%	9%	9%	2%	1%	44%	3%	1%	0%	26%
	**Range**	0–10%	0–8%	6–14%	0–31%	0–6%	0–3%	19–59%	0–8%	0–6%	0–6%	6–50%

### Planned birth practices: inter-disciplinary variation

Comparison between responses from midwives and medical staff identified significant variation in relation to perceptions around women’s involvement in decision-making for all four items. Midwives were consistently less likely to agree or strongly agree that women are engaged in decision-making about their own care (See Table 2). For example, only 68% of midwives agreed or strongly agreed that “Women are informed about the benefits and risks of interventions such as IOL and CS” versus 93% of medical staff.

While less pronounced than the different perceptions around women’s involvement in decision-making, there was also statistically significant variation between how midwives and medical staff rated 8 out of 18 statements in relation to indications for planned birth. The statements with the most inter-disciplinary variation (20% or more) were:

- Women with uncomplicated MCDA twin pregnancies, where the presenting twin is cephalic, are supported to have a vaginal birth (50% of midwives versus 73% of medical staff agreed or strongly agreed).
- Women who are afraid of childbirth are counselled and provided with information about the pros and cons of CS (64% of midwives versus 84% of medical staff agreed or strongly agreed).
- Women with an uncomplicated breech (frank or complete breech, normal fetal size and welfare) are supported to have a vaginal birth, either in our unit or by referral to a unit which offers vaginal breech birth (45% versus 65%)

No comparison was made between the timing of IOL responses from midwives versus medical staff because of the small subgroup numbers.

### Readiness for change in relation to planned birth

As indicated in Table 5, on average the readiness for change of the participating units was poor, with considerable variation in the culture of different units. There was statistically significant inter- hospital variation for 11 out of the 14 items.

**Table 5:** Organisational readiness for change in relation to planned birth - % of respondents who agreed or strongly agreed with the statement

	% of all respondents	Hospital A, level 6	Hospital B, level 6	Hospital C, level 5	Hospital D, level 4	Hospital E, level 4	Hospital F, level 4	Hospital G, level 3	***p***-value	Midwives	Medical staff	***p***-value
We are a real team, we understand and respect roles and expertise	73%	81%	61%	86%	75%	64%	92%	44%	.011*	69%	85%	.120
Our leaders are visible and vocal	69%	73%	51%	76%	82%	54%	93%	50%	< 0.001*	65%	80%	.200
Our guidelines are evidence-based and up-to-date	73%	81%	55%	87%	94%	58%	93%	76%	< 0.001*	71%	81%	.005*
We all practice to the same guidelines – no opting out	47%	46%	16%	79%	56%	32%	92%	37%	< 0.001*	43%	57%	.098
We get accurate, timely, relevant information on our performance	42%	34%	23%	55%	57%	40%	62%	37%	.005*	40%	50%	.474
We get relevant information that benchmarks our performance against other maternity units	37%	29%	26%	31%	51%	40%	54%	44%	.728	33%	50%	.152
There is a need to change our practices in relation to planned CS to improve outcomes for women and babies	48%	31%	64%	48%	26%	93%	38%	56%	< 0.001*	54%	26%	< 0.001*
There is a need to change our practices in relation to IOL to improve outcomes for women and babies	64%	50%	83%	52%	57%	89%	39%	75%	< 0.001*	70%	43%	< 0.001*
Senior leadership reward clinical innovation and creativity to improve care	33%	42%	6%	44%	57%	46%	38%	25%	.067	29%	47%	.804
Senior leadership provide effective management for continuous improvement of care	45%	46%	19%	69%	79%	36%	46%	37%	.048*	42%	58%	.489
Senior leadership hold staff members accountable for achieving results	39%	48%	16%	44%	51%	43%	38%	32%	.733	35%	52%	.742
In general, when there is agreement that change needs to happen we have the necessary support in terms of budget or financial resources	19%	17%	6%	37%	19%	21%	23%	19%	.031*	16%	34%	.030*
In general, when there is agreement that change needs to happen we have the necessary support in terms of training	48%	52%	26%	69%	57%	57%	46%	44%	.023*	44%	64%	.231
In general, when there is agreement that change needs to happen we have the necessary support in terms of staffing	23%	27%	6%	44%	25%	18%	15%	25%	.001*	18%	42%	.003*

*statistical significance at < 0.05

Regarding the need for change, 48% agreed “there is a need to change practices in relation to planned CS” (range 26–93%). On average, 64% agreed “there is a need to change our practices in relation to IOL to improve outcomes for women and babies” (range 39–89%). In terms of the culture of the unit to enable change, while an average 73% of respondents agreed or strongly agreed that they worked as a team and understand and respect roles and expertise, this ranged from 44 to 92% between units. While 69% agreed that their leaders were visible and vocal, this ranged from 50 to 93%. A total of 73% agreed that guidelines are evidence-based and up-to-date (range 55–94%) but only 49% agreed that they practice to the same guidelines (range 16–92%) and only 42% agreed that they get accurate, timely, relevant performance information (range 23–62%). Less than half (average 45%, range 19–79%) agreed that senior leadership provide effective management for continuous improvement of care.

In terms of the supports available for necessary change, only 19% agreed that the required budget or financial resources were available (range 6–37%), 48% agreed that the necessary support in terms of training was available (range 26–69%), and 23% agreed the necessary support in terms of staffing was available (range 6–44%).

Despite considerable range between hospitals, there was no significant difference between units in relation to the availability of benchmarking data (37%, range 26–54%), the availability of support for clinical innovation and creativity to improve care from senior leadership (33%, range 6 to 46%), and whether staff members are held accountable for achieving results (39%, range from 16 to 51%).

There was greater consistency in perceptions around readiness for change between disciplines, with significant variation between midwifery and medical staff for only 5 items. However, the differences seen are notable in that medical staff were less likely to see a need for change in either CS practices (26% versus 56% midwives) or IOL practices (43% versus 70% of midwives). Medical staff were also more likely to agree that guidelines are evidence-based and up-to-date (81% versus 71% of midwives), and to agree that necessary financial support (34% versus 16%) and staffing support (42% versus 18%) is available to support required change.

The other notable finding related to readiness for change was a mismatch between perceived need and ability. Specifically, the three hospitals (B, E and G) where respondents were mostly likely to agree change in CS practices was needed (64, 93, 56% respectively – average 48% all hospitals) and change in IOL practices was needed (83, 89, 75% respectively – average 64%), also perceived they were resource poor. In these hospitals, only 6, 21 and 19% agreed financial resources were available to support change, while only 6, 18 and 25% agreed staffing resources were available. There was also on average less agreement on the strength of teams, leaders and guidelines at these hospitals, particularly Hospital B (Level 6).

## Discussion

This study found significant inter-hospital and inter-professional variation in perceptions of hospital planned birth practices, and requirement for and readiness for change of such practices. While some of this variation reflects genuine uncertainty in the literature regarding a particular planned birth indication (e.g. IOL for mothers of South-Asian origin), there was also variation in practices where national and/or international guidelines offer specific evidence-based guidance e.g. preeclampsia at 37+ weeks, uncomplicated breech presentation [31, 32]. There was also considerable variation in perception of women’s involvement in decision-making both between units and disciplines, with midwifery staff less likely to agree women were appropriately informed and involved in decisions. Of concern for the prospects of improvements to planned birth practices in the participating hospitals, we also found that the units where staff most often perceived change was needed were also those units where resources of leadership, guidelines, staff and overall finances to support change were seen as less available.

Women’s engagement in decision-making was perceived much more positively in some hospitals than others. There was also inter-disciplinary variation, with midwifery staff less likely to agree that women were appropriately informed about planned birth and engaged in decision-making. Our finding that the level of agreement that women are informed about the benefits and risks of interventions such as IOL and CS ranged from 22 to 95% indicates that best-practice guidelines in relation to women’s involvement in decisions about their own care [33, 34] are not routinely implemented in practice across the study hospitals. This also highlights gaps between theory and practice, as in our previously reported findings from these units over 99% of both midwives and medical practitioners agreed that women should be informed of risks and benefits of IOL and planned CS, and 97 and 91% of midwives and medical practitioners respectively agreed that women should be supported to make decisions about their own care [23].

Inter-hospital variation in perception of indications for planned birth also highlights inconsistent application of evidence-based guidelines. While some variation can be partially explained by the different hospital levels (e.g. lower level hospitals were less likely to provide intrapartum care for women with twin pregnancies, breech pregnancies, and previous shoulder dystocia), some variation in planned birth practices remain unexplained. For example, over a quarter of respondents at 4 of the 7 hospitals agreed that women with abnormal fetal lie (e.g. breech or transverse) are routinely offered a CS in preference to External Cephalic Version (ECV), in conflict with RANZCOG evidence-informed guidance that ECV should be offered to all women in whom it is appropriate [32]. This variation in *perceived* practices around the provision of planned birth was evident in the *actual* rates of IOL and prelabour CS in an analysis of routinely collected hospital data conducted in a related study [35]. Even after adjusting for case-mix factors, there was substantial variation in the rates of IOL (27.6–42%) and prelabour CS (15.4–22.6%) across the participating hospitals [35].

Variation in timing of planned birth also indicates a lack of consistent evidence-based care. This was seen both with planned birth earlier than recommended, for example up to 31% at one site agreeing it is common that “women with uncomplicated pregnancies are offered IOL before 41+0 weeks” and up to 37% agreeing planned CS (without indication for earlier birth) is offered before 39 + 0 weeks, as well as not offering IOL or offering it later than guidelines recommend. Regarding preeclampsia, both the ISSSHP [36] and the SOMANZ [31] guidelines recommend birth at 37 weeks (or promptly after diagnosis if diagnosis made > 37 weeks) so it is of some concern that over two-thirds of respondents did not nominate 37 weeks as when IOL would be offered from, and 16% agreed that preeclampsia would only be an indication for induction from 40 weeks, 41 weeks, or not at all.

In relation to PROM, 14% of respondents indicated term PROM was not an indication for IOL for GBS-positive women, and only 35% of respondents indicated that GBS-negative women were offered IOL by 24 h, despite RANZCOG guidelines that suggest even if IOL is not immediately offered for term PROM, there should only be “a short trial of expectant management (e.g. for up to 24/24) in highly selected and well supervised cases” [37]. (p. 6). This is in contrast with findings by Blanc-Petitjean et al. (2018) who studied planned birth practices in 94 maternity units in France [38] and found that after PROM for GBS-negative women, the maximum delay before IOL was 24 h for 58.5% of the maternity units and 36 to 48 h for 36.2%. If the vaginal sample was GBS-positive, nearly one third reported that they induced labour immediately (29.8%) and more than 90% within 24 h [38].

Other inter-hospital variations are reflective of different policies between hospitals rather than inconsistent application of guidelines. For example, two of the participating hospitals have a policy to induce women from some ethnic groups, such as South Asian women, at earlier gestations (based on epidemiological evidence of higher rates of term stillbirth in these women [39]), while other hospitals in the absence of higher-level evidence such as an RCT have not adopted such a policy. Similarly, variation in relation to vaginal birth after CS reflect that some units do not offer vaginal births after a previous CS.

Our finding that there is considerable inter-hospital variation is consistent with findings by Blanc-Petitjean et al. (2018) which found considerable variation in IOL practices between the 94 French maternity units studied. This study found variation in IOL practices, with only 28.7% of units inducing labour for breech presentation, 70.2% for a previous caesarean, 45.7% for suspected fetal macrosomia without diabetes and 63.8% for fetal growth restriction. Inter-hospital variation in indications for IOL is not surprising given the considerable uncertainty in the literature and clinical guidelines around what constitutes best-practice [17, 18, 40]. Variation may relate to uncertainty and confusion in the literature about when to recommend IOL and the consequences of IOL at that specific gestation for the particular indication of IOL [40]. While it was traditionally thought that IOL increased the risk of CS, recent evidence suggests otherwise [41], and there remains considerable debate around the circumstances in which IOL should be offered.

This study also found significant inter-professional variation, indicating disagreement between midwives and medical staff on how they view their hospital practices. Few statements had equal support from both professional groups in the way their units practiced. In particular, we found a disconnect in perceptions around women’s involvement in decision-making, with midwives consistently less likely to agree or strongly agree that women were engaged in decisions about their own care. Before an IOL or planned CS is conducted, most women require a consultation with medical staff to discuss the need for intervention. While midwives may have discussions with women about the need for a planned birth when circumstances arise, ultimately the responsibility lies with medical staff to recommend a course of action in consultation with the woman. As such, our findings reflect, at least in part, a midwifery perception of deficiency in counselling and shared decision-making by their medical colleagues. The medical staff themselves did not perceive this to be an issue. These findings are consistent with studies that have shown inter-professional variation in attitudes towards birth and women’s engagement in decision-making [20, 42], with different attitudes informing variation in practice [43, 44]. Findings from a related study conducted by the authors provide insight into why variation occurs from the perspective of clinicians at these hospitals, highlighting the role of inconsistency and ambiguity in local guidelines, policies and procedures; contradictory research and different interpretations of evidence; clinician preferences, beliefs and values; the culture of the unit; and organisational influences such as access to specialised clinics and theatre time [35].

The identification of variation in clinical practice is only the first step to addressing this evidence-practice gap. Our study also highlighted several issues relating to readiness for change, a key facilitator or barrier to closing that gap. We found both inter-hospital and inter-professional differences in perceptions of the readiness of units to make changes in relation to planned birth practices, with medical staff more likely to rate the culture and readiness for change of their unit more favourably and less likely to perceive a need for change compared to midwives. In addition, we found significant difference in perceptions of readiness for change between units, with those three units where respondents indicated change was required, least change ready. Despite variation between units, all units were perceived to experience shared barriers to change, particularly a lack of access to performance information as well as limited access to resources when change is needed. However, it is concerning regarding potential for practice change that those units who perceived greatest need are also those reporting the fewest resources in terms of culture and personnel to do so. The results of this study were shared with the participating hospitals to encourage quality improvement initiatives that may close the evidence to practice gap. Informed by the finding that women are not consistently engaged in decisions about their own care, one initiative currently underway is training in shared decision-making for both medical and midwifery staff.

### Strengths and limitations

A strength of this study is its assessment of staff perceptions of current practice and of what needs to change, including perceptions of what resources are available to support change. Coupled with our previous findings [23], it also highlights variation between expressed theoretical beliefs (for example regarding women’s involvement in planned birth decision-making) and belief about what is happening in their unit’s current practice. Weaknesses include the modest sample size, and the consequent lack of controlling for inter-hospital responses by discipline and other potentially important factors such as gender (due to small subgroup numbers). The distribution of midwives and medical staff (who had disparate responses on many items) was not consistent across hospitals which could explain differences in hospital by hospital response, and not controlling for this is a limitation. However, it should be noted that inter-disciplinary variation alone would not explain inter-hospital variation (given no hospital had more than a third of responses from their medical staff, nor any under 10%), and hospitals with a relatively higher proportion of responses from medical staff did not necessarily rate their performance more highly. Another limitation is the survey response rate of approximately 27%. Although comparable to the response rate of other surveys of maternity care providers in Australia [45, 46], self-selection bias means those who have more strongly held views around planned birth and unit performance may be more likely to participate. Without hearing from less engaged clinicians we cannot know what their views might be.

## Conclusion

Few studies have assessed the uptake of evidence in maternity units in Australia, and our study demonstrates variation in the uptake of evidence-based guidelines between hospitals. Our findings are consistent with previous observations that evidence is not routinely implemented in maternity care, both in Australia and overseas. More specifically, our results show significant variation between the units on both their beliefs about their units’ practices, some of which conflict with the evidence, and their readiness to change. Additionally, hospitals with greatest staff perception of need for change also perceived least resources to implement such change, underscoring potential barriers to change in planned birth care. Our results also show that there is significant variation and even conflict between the understanding and practice of the two professional groups, and an indication that medical staff are less likely to perceive a need for their unit to make changes to their current practice around planned birth. This study highlights some of the reasons contributing to variation in clinical practice as well as challenges and potential barriers to reducing the unwarranted clinical variation that currently exists across the participating hospitals.

## Supplementary Information

**Additional file 1: Supplementary file 1**. Clinician Survey – for midwives and obstetricians.

**Additional file 2.**

## Data Availability

The data is available upon request to the corresponding author.
