# Supplementary material for: Inter-hospital and inter-disciplinary variation in planned birth practices and readiness for change: a survey study
Source: BMC Pregnancy Childbirth. 2021 May 20;21:391. doi: 10.1186/s12884-021-03844-z (PMC8135152; doi:10.1186/s12884-021-03844-z)
Supplement: Supplementary file 1 — Additional file 1: Supplementary file 1. Clinician Survey – for midwives and obstetricians. [file 12884_2021_3844_MOESM1_ESM.docx]

**Supplementary file 1: Clinician Survey – for midwives and obstetricians**

**Inter-hospital and inter-disciplinary variation in planned birth practices and readiness for change: a survey study**

Coates, Dominiek^1, 2^ (Corresponding author) Correspondence to [Dominiek.Coates@uts.edu.au](mailto:Dominiek.Coates@uts.edu.au)

Donnolley, Natasha^4^

Foureur, Maralyn^8, 5^

Henry, Amanda^3, 6, 7^

^1^University of Technology Sydney, Faculty of Health, Centre for Midwifery and Child and Family Health

^2^Maridulu Budyari Gumal, the Sydney Partnership for Health, Education, Research and Enterprise (SPHERE)

^3^ School of Women's and Children's Health, UNSW Medicine, UNSW, Sydney, Australia

^4^ National Perinatal Epidemiology and Statistics Unit, Centre for Big Data Research in Health, UNSW, Sydney, Australia

^5^University of Newcastle, Faculty of Health and Medicine, Australia

^6^ Department of Women's and Children's Health, St George Hospital, Sydney, Australia

^7^ The George Institute for Global Health, UNSW Medicine, Australia

^8^Hunter New England Nursing and Midwifery Research Centre, Australia

**Clinician Survey – for midwives and obstetricians**

**Demographic Questions**

For each question, please indicate the single most correct option.

My discipline/role:

- Midwife:
  - Primary area:
    - Antenatal care
    - Postnatal care
    - Intrapartum care
    - Midwifery Group Practice
    - CMC, CMS or CME
    - Management
    - All areas

Obstetrician, work predominantly public

Obstetrician, work equal public and private

- Obstetrician, work predominantly private
- Obstetric Registrar/Resident

My sex:

- Female
- Male
- Non-binary
- Prefer not to say

My age:

- 20-30
- 31-40
- 41-50
- 51-60
- Over 60
- Prefer not to say

My years of experience:

- Less than 5
- Between 5 and 10
- Between 11 and 15
- 16 or more
- Prefer not to say

My primary affiliated public hospital:

- Options blinded for confidentiality

**practices in our unit**

This section contains statements about practices in relation to IOL and CS **in your unit.** While we recognise that practice is not black and white, make your choice by selecting the response that most accurately reflects the practices in your unit. For questions in relation to IOL, assume no prior CS or relevant contraindication to a vaginal birth. **IN OUR UNIT ….**

|  | **Strongly disagree** | **Disagree** | **Undecided** | **Agree** | **Strongly agree** |
| --- | --- | --- | --- | --- | --- |
| Women are informed about the benefits and risks of interventions such as IOL and CS |  |  |  |  |  |
| Women are supported to make decisions about their own care in relation to IOL |  |  |  |  |  |
| Women are supported to make decisions about their own care in relation to CS |  |  |  |  |  |
| Consideration is given to the wishes and preferences of the woman in decisions about her care in relation to IOL and CS |  |  |  |  |  |
| In terms of the management of prolonged pregnancy, women from some ethnic groups, such as South Asian women, are induced at earlier gestations |  |  |  |  |  |
| Women with uncomplicated DCDA twin pregnancies, where the presenting twin is cephalic, are supported to have a vaginal birth |  |  |  |  |  |
| Women with uncomplicated MCDA twin pregnancies, where the presenting twin is cephalic, are supported to have a vaginal birth |  |  |  |  |  |
| Women who are afraid of childbirth are counselled and provided with information about the pros and cons of CS |  |  |  |  |  |
| Women who request a CS (without a medical reason) are counselled and supported to have a vaginal birth |  |  |  |  |  |
| Women who had a previous CS are supported to have a vaginal birth |  |  |  |  |  |
| Women with an uncomplicated breech (frank or complete breech, normal fetal size and welfare) are supported to have a vaginal birth, either in our unit or by referral to a unit which offers vaginal breech birth |  |  |  |  |  |
| Women who had a previous uterine rupture are routinely recommended a CS |  |  |  |  |  |
| Women with abnormal fetal lie (e.g. transverse lie), where ECV is declined, unsuccessful or not appropriate, are routinely recommended a CS |  |  |  |  |  |
| Women with abnormal fetal lie (e.g. breech or transverse) are routinely offered a CS in preference to ECV |  |  |  |  |  |
| Women with prior classical or inverted T uterine incision are recommended a repeat CS |  |  |  |  |  |
| Women with previous perineal trauma (e.g. 3rd or 4th degree tear/obstetric anal sphincter injury) are offered a CS |  |  |  |  |  |
| Women with previous severe pelvic floor damage (e.g. prolapse) are offered a CS |  |  |  |  |  |
| Women with a previous fetal death in utero are routinely offered a CS |  |  |  |  |  |
| Women with previous shoulder dystocia are routinely offered a CS |  |  |  |  |  |
| Women who are colonised with Group B Strep are offered a CS |  |  |  |  |  |

In our unit, women with uncomplicated pregnancies are offered IOL:

- Before 39 completed weeks
- From 39+0-39+6 weeks onwards
- From 40+0-40+6 weeks onwards
- From 41+0-41+6 weeks onwards
- From 42+0 weeks onwards

In our unit, planned CS for singleton pregnancy where there is no maternal or fetal indication for early birth, are offered from:

- 35+0 weeks onwards
- 36+0 weeks onwards
- 37+0 weeks onwards
- 38+0 weeks onwards
- 39+0 weeks onwards
- 40+0 weeks onwards

Please rate the statements below about practices in your unit in relation to the timing of **IOL.** Please choose the single most correct option, assuming for each indication that it is the only reason for IOL, and that the woman does not have a contraindication to vaginal birth. Please note, the “not a reason for IOL” option does not mean women in your unit are *never* induced for this reason: it indicates that it is not standard practice to induce for this reason/IOL for this reason would only be on a case by case basis. **IN OUR UNIT (public hospital), women with the conditions below are induced at:**

|  | **35+0 weeks onwards** | **36+0 weeks onwards** | **37+0 weeks onwards** | **38+0 weeks onwards** | **39+0 weeks onwards** | **40+0 week onwards** | **41+0 weeks onwards** | **not a reason for IOL** |
| --- | --- | --- | --- | --- | --- | --- | --- | --- |
| Suspected macrosomia |  |  |  |  |  |  |  |  |
| Request for IOL without medical indications |  |  |  |  |  |  |  |  |
| Gestational diabetes that is diet controlled |  |  |  |  |  |  |  |  |
| Gestational diabetes managed with oral hypoglycaemics (e.g. metformin) |  |  |  |  |  |  |  |  |
| Insulin-requiring gestational diabetes (not pre-pregnancy Type I or II) |  |  |  |  |  |  |  |  |
| Pre-pregnancy diabetes, Type I |  |  |  |  |  |  |  |  |
| Pre-pregnancy diabetes, Type II |  |  |  |  |  |  |  |  |
| Gestational hypertension (new-onset high blood pressure after 20 weeks, no preeclampsia) |  |  |  |  |  |  |  |  |
| Chronic/essential hypertension |  |  |  |  |  |  |  |  |
| Preeclampsia (assume no urgent indication for birth) |  |  |  |  |  |  |  |  |
| Uncomplicated monochorionic diamniotic (MCDA) twin pregnancies (if vaginal birth is planned) |  |  |  |  |  |  |  |  |
| Uncomplicated dichorionic diamniotic (DCDA) twin pregnancies (if vaginal birth is planned) |  |  |  |  |  |  |  |  |
| Cholestasis of pregnancy |  |  |  |  |  |  |  |  |
| Maternal age of 40 and over |  |  |  |  |  |  |  |  |
| Substantially elevated BMI (> 40 kg/m2) |  |  |  |  |  |  |  |  |

Please rate the statements below about practices in your unit in relation to the timing of **IOL for premature rupture of membranes (PROM).** In relation to the timing of IOL, **in our unit** (public hospital), women with no other complications or signs of chorioamnionitis, with PROM:

|  | **ASAP** | **within 12 hours** | **13-24 hours** | **25-48 hours** | **49-96 hours** | **After 96 hours** | **At 37+0 weeks** | **At 38+0 weeks** | **At 39+0 weeks** | **At 40+0 weeks** | **Not induced** |
| --- | --- | --- | --- | --- | --- | --- | --- | --- | --- | --- | --- |
| A**t term** (37+0) who are Group B streptococcus positive |  |  |  |  |  |  |  |  |  |  |  |
| A**t term** (>37+0) who are Group B streptococcus negative |  |  |  |  |  |  |  |  |  |  |  |
| **Late preterm** (34+0 - 36+6 weeks) who are Group B streptococcus positive |  |  |  |  |  |  |  |  |  |  |  |
| **Late preterm** (34+0-36+6 weeks) who are Group B streptococcus negative |  |  |  |  |  |  |  |  |  |  |  |

**Section 4: Organisational readiness for change**

This section contains statements about the readiness for change in your unit. Please rate the following questions from strongly agree to strongly disagree to best reflect your opinion about **your unit. IN OUR UNIT…**

|  | **Strongly disagree** | **Disagree** | **Neutral** | **Agree** | **Strongly agree** | **I don’t know** |
| --- | --- | --- | --- | --- | --- | --- |
| We are a real team, we understand and respect roles and expertise |  |  |  |  |  |  |
| Our leaders are visible and vocal |  |  |  |  |  |  |
| Our guidelines are evidence-based and up-to-date |  |  |  |  |  |  |
| We all practice to the same guidelines – no opting out |  |  |  |  |  |  |
| We get accurate, timely, relevant information on our performance |  |  |  |  |  |  |
| We get relevant information that benchmarks our performance against other maternity units |  |  |  |  |  |  |
| There is a need to change our practices in relation to planned CS to improve outcomes for women and babies |  |  |  |  |  |  |
| There is a need to change our practices in relation to IOL to improve outcomes for women and babies |  |  |  |  |  |  |
| Senior leadership reward clinical innovation and creativity to improve care |  |  |  |  |  |  |
| Senior leadership provide effective management for continuous improvement of care |  |  |  |  |  |  |
| Senior leadership hold staff members accountable for achieving results |  |  |  |  |  |  |
| In general, when there is agreement that change needs to happen we have the necessary support in terms of budget or financial resources |  |  |  |  |  |  |
| In general, when there is agreement that change needs to happen we have the necessary support in terms of training |  |  |  |  |  |  |
| In general, when there is agreement that change needs to happen we have the necessary support in terms of staffing |  |  |  |  |  |  |
