# Supplementary material for: Inter-hospital and inter-disciplinary variation in planned birth practices and readiness for change: a survey study
Source: BMC Pregnancy Childbirth. 2021 May 20;21:391. doi: 10.1186/s12884-021-03844-z (PMC8135152; doi:10.1186/s12884-021-03844-z)
Supplement: Supplementary file 2 — Additional file 2. [file 12884_2021_3844_MOESM2_ESM.docx]

**Supplementary file 2**

**Inter-hospital and inter-disciplinary variation in planned birth practices and readiness for change: a survey study**

Coates, Dominiek^1, 2^ (Corresponding author) Correspondence to [Dominiek.Coates@uts.edu.au](mailto:Dominiek.Coates@uts.edu.au)

Donnolley, Natasha^4^

Foureur, Maralyn^8, 5^

Henry, Amanda^3, 6, 7^

^1^University of Technology Sydney, Faculty of Health, Centre for Midwifery and Child and Family Health

^2^Maridulu Budyari Gumal, the Sydney Partnership for Health, Education, Research and Enterprise (SPHERE)

^3^ School of Women's and Children's Health, UNSW Medicine, UNSW, Sydney, Australia

^4^ National Perinatal Epidemiology and Statistics Unit, Centre for Big Data Research in Health, UNSW, Sydney, Australia

^5^University of Newcastle, Faculty of Health and Medicine, Australia

^6^ Department of Women's and Children's Health, St George Hospital, Sydney, Australia

^7^ The George Institute for Global Health, UNSW Medicine, Australia

^8^Hunter New England Nursing and Midwifery Research Centre, Australia

**Supplementary figures to Table 3**

Survey respondents’ perceptions of practices in relation to the timing of IOL – average and range of responses between units

*Respondents were asked to choose the single most correct option, assuming for each indication that it is the only reason for IOL, and that the woman does not have a contraindication to vaginal birth. Please note, the “not a reason for IOL” option does not mean women in your unit are never induced for this reason: it indicates that it is not standard practice to induce for this reason/IOL for this reason would only be on a case by case basis.*

Suspected macrosomia

**
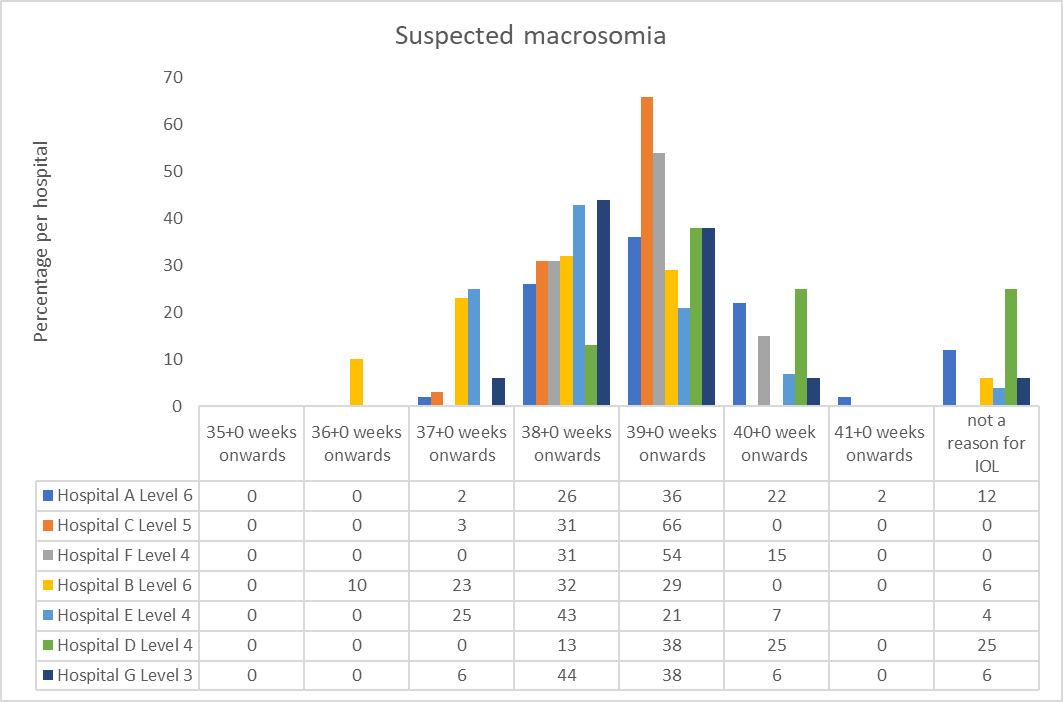
**

Request for IOL without medical indications

**
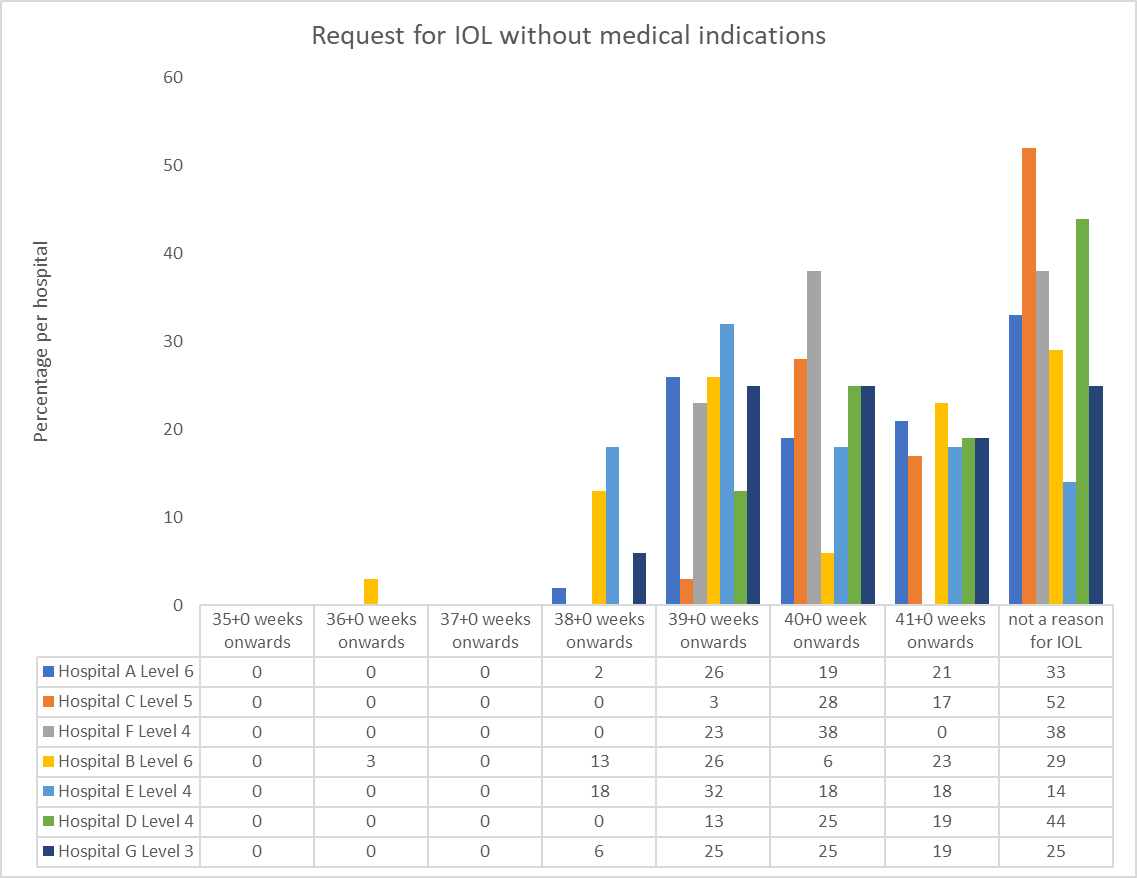
**

Gestational diabetes that is diet controlled

**
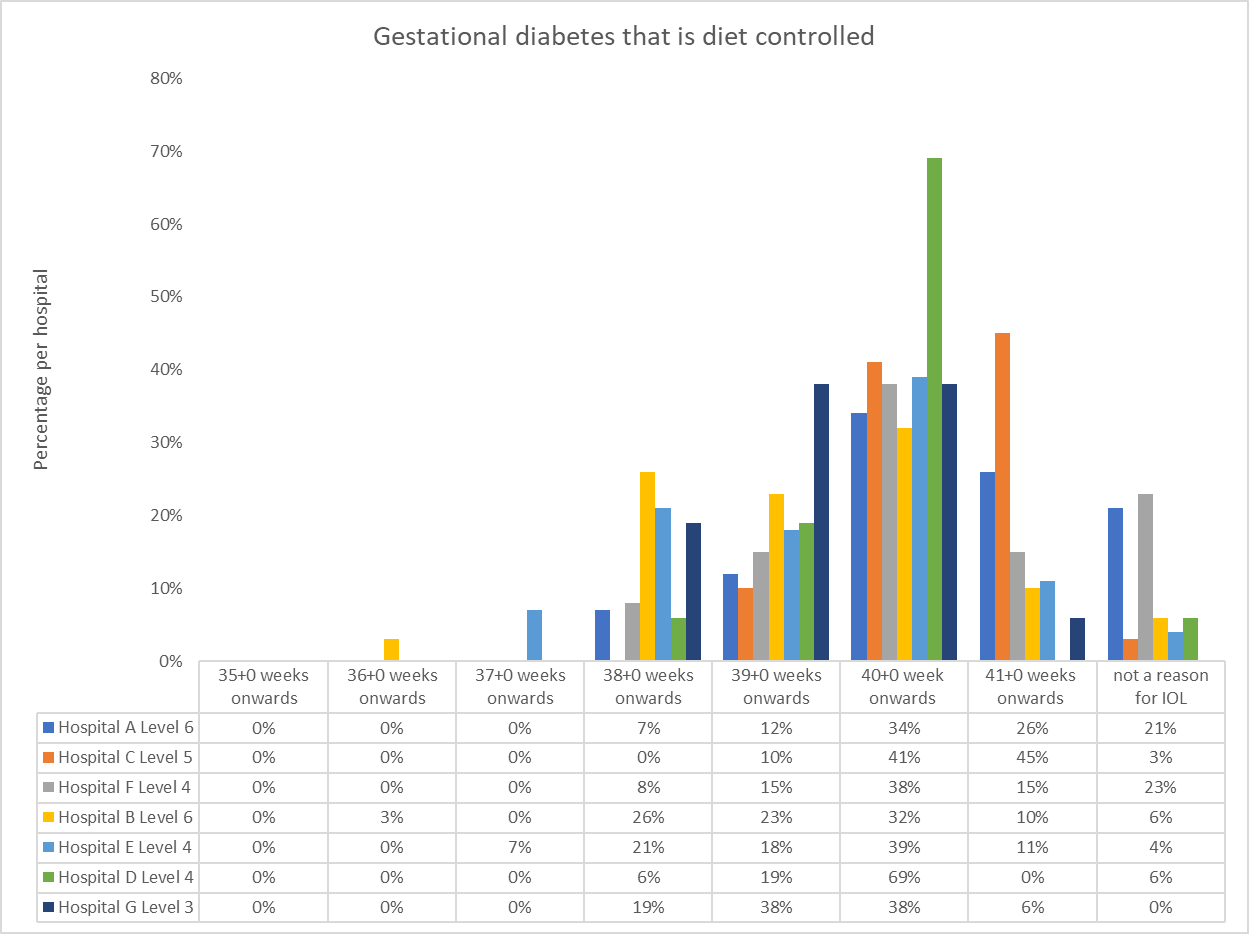
**

Gestational diabetes managed with oral hypoglycaemics (e.g. metformin)

**
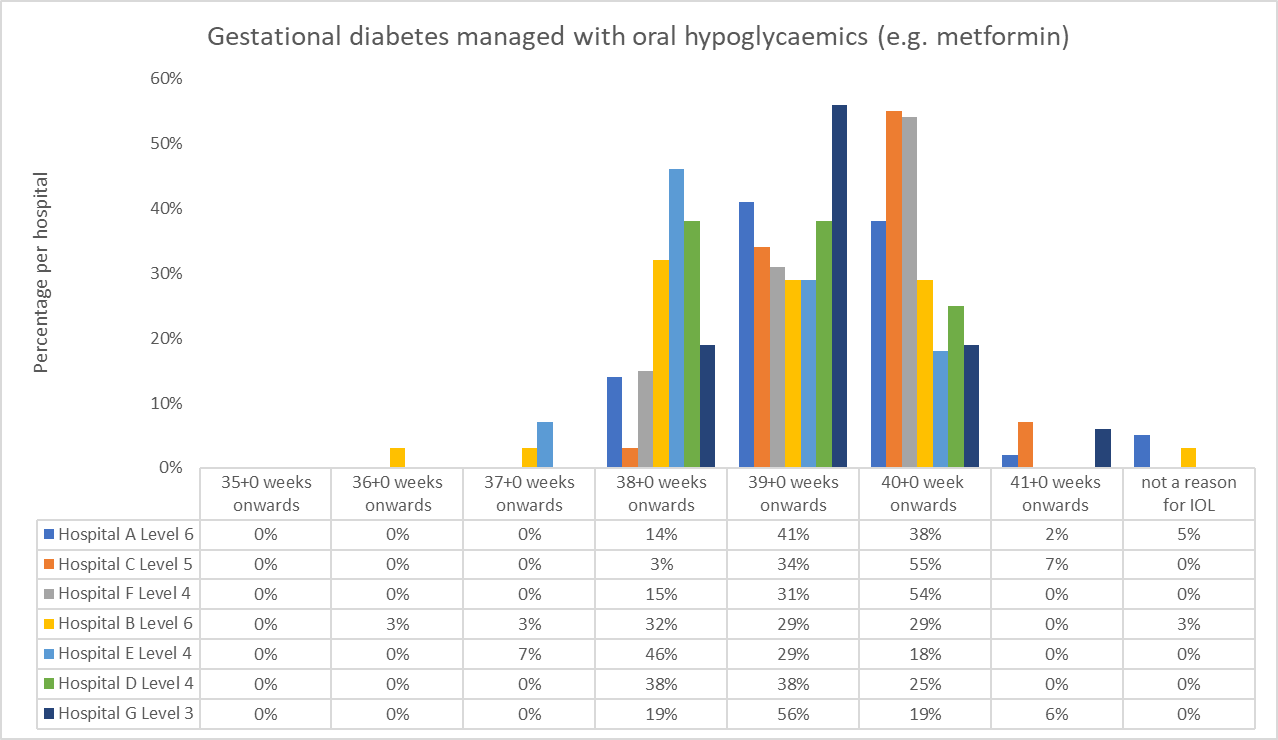
**

Insulin-dependent gestational diabetes (not pre-pregnancy Type I or II)

**
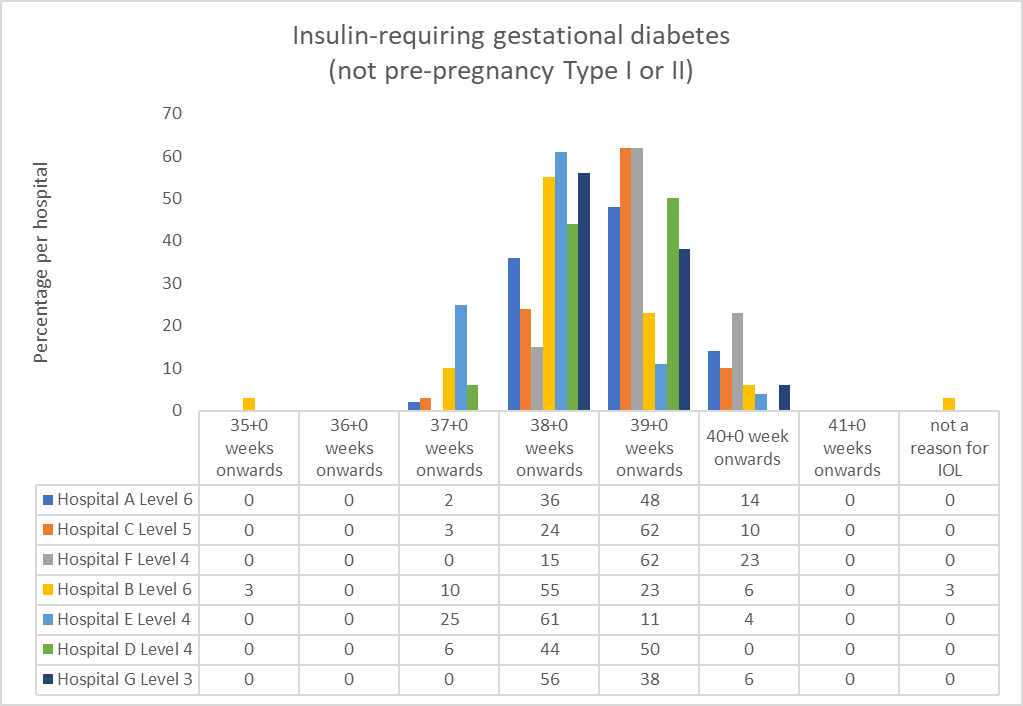
**

Pre-pregnancy diabetes, Type I

**
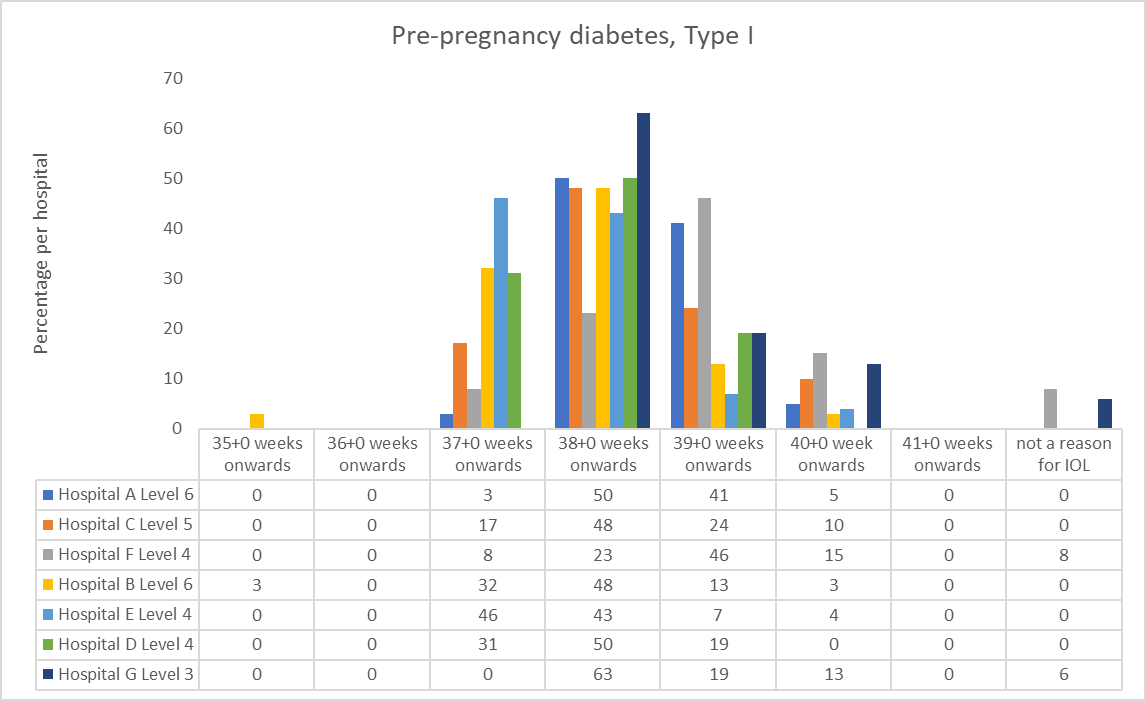
**

Pre-pregnancy diabetes, Type II

**
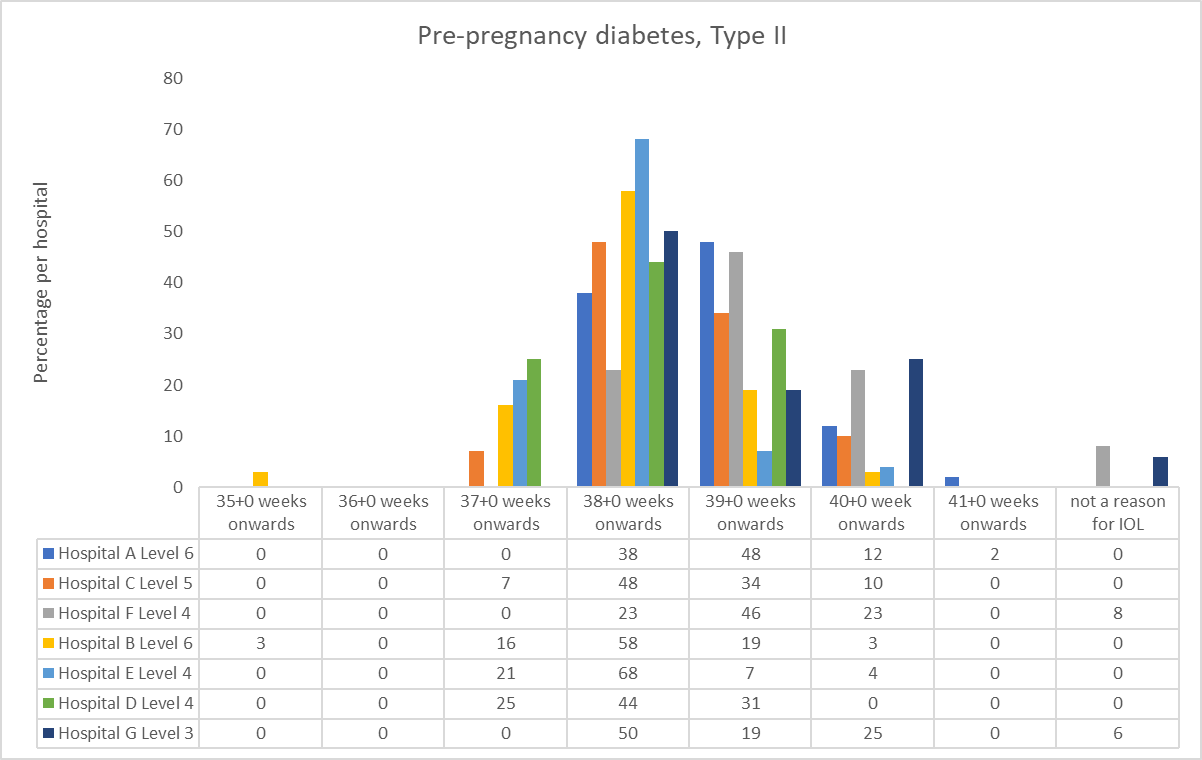
**

Gestational hypertension (new-onset high blood pressure after 20 weeks, no preeclampsia)

**
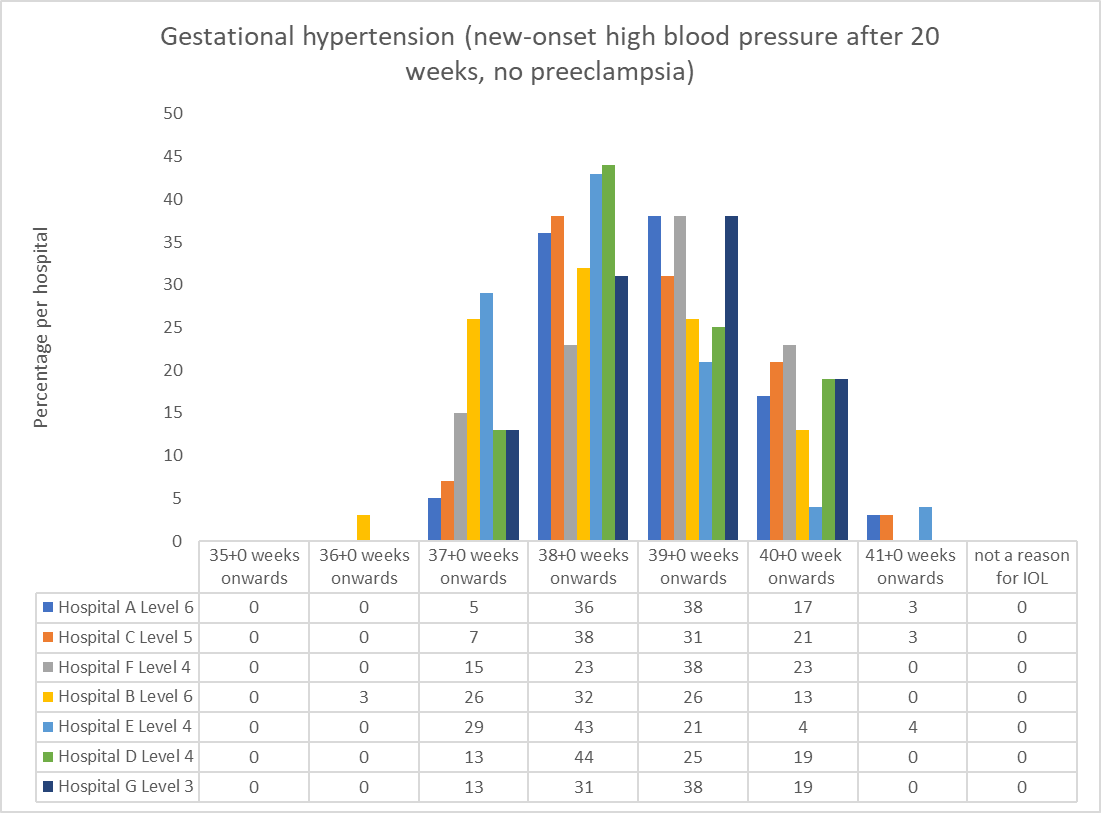
**

Chronic/essential hypertension

**
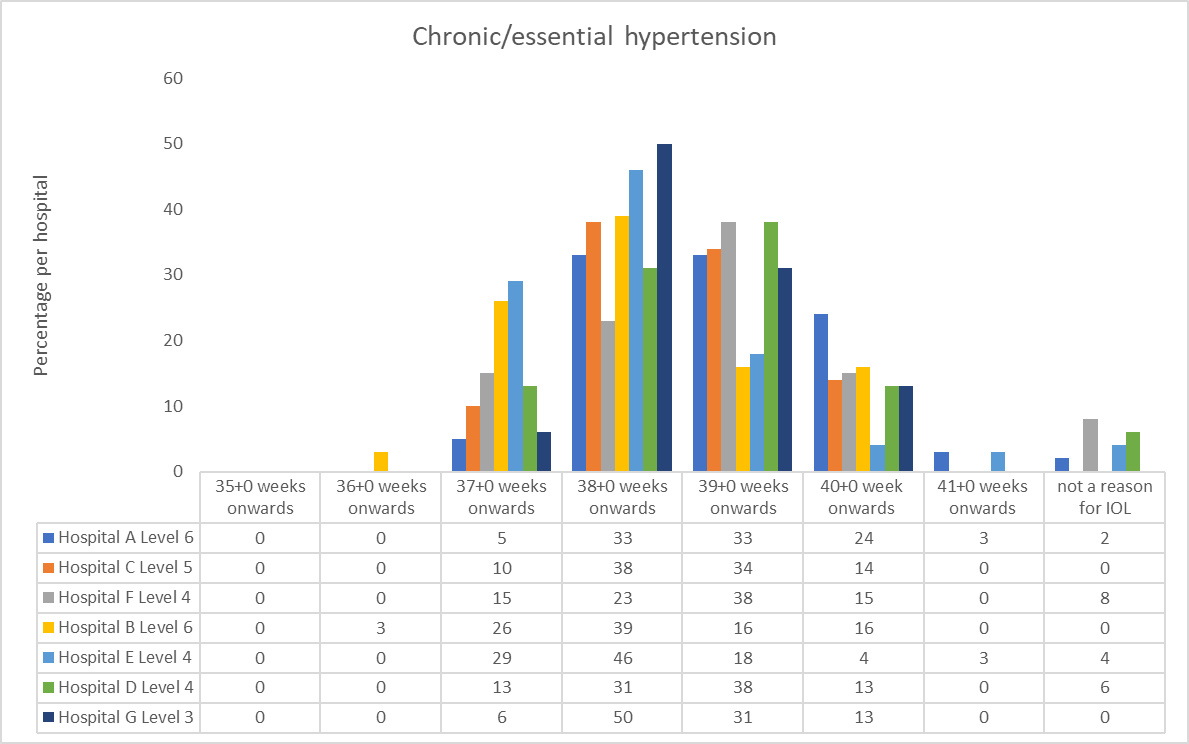
**

Preeclampsia (assume no urgent indication for birth)

**
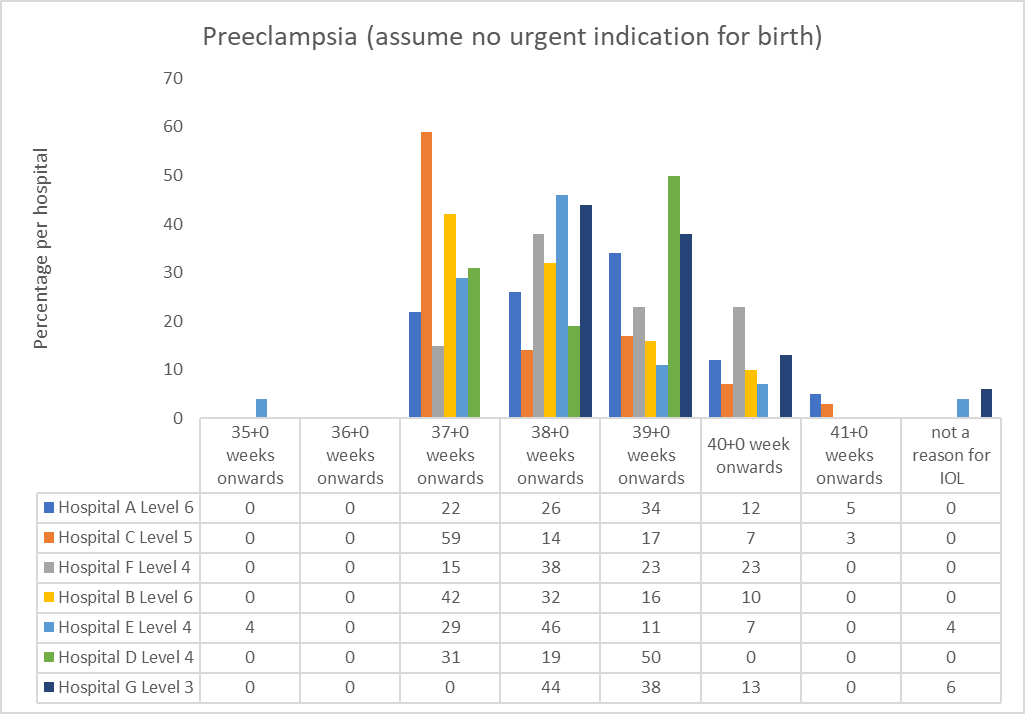
**

Uncomplicated monochorionic diamniotic (MCDA) twin pregnancies (if vaginal birth is planned)

**
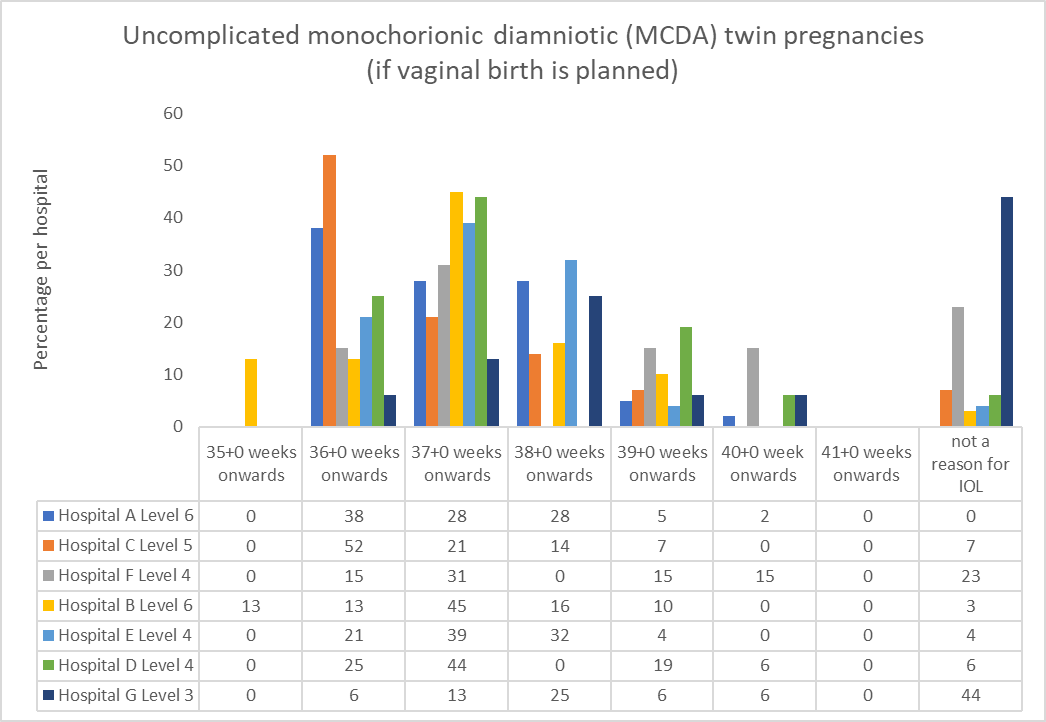
**

Uncomplicated dichorionic diamniotic (DCDA) twin pregnancies (if vaginal birth is planned)

**
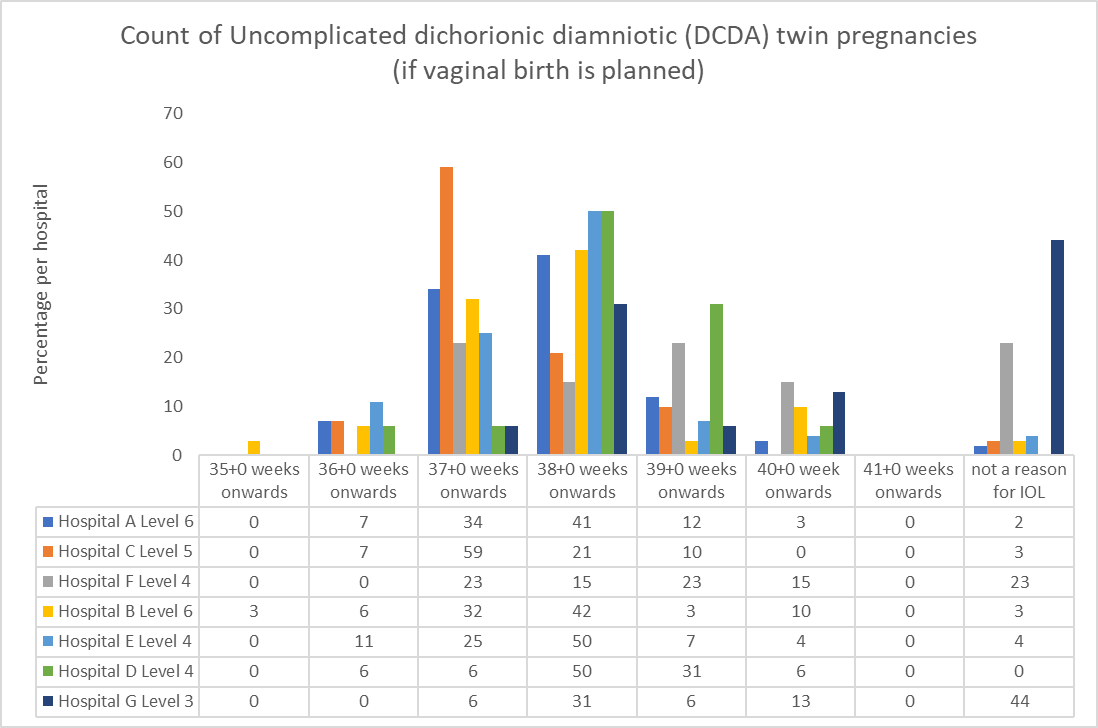
**

Cholestasis of pregnancy

**
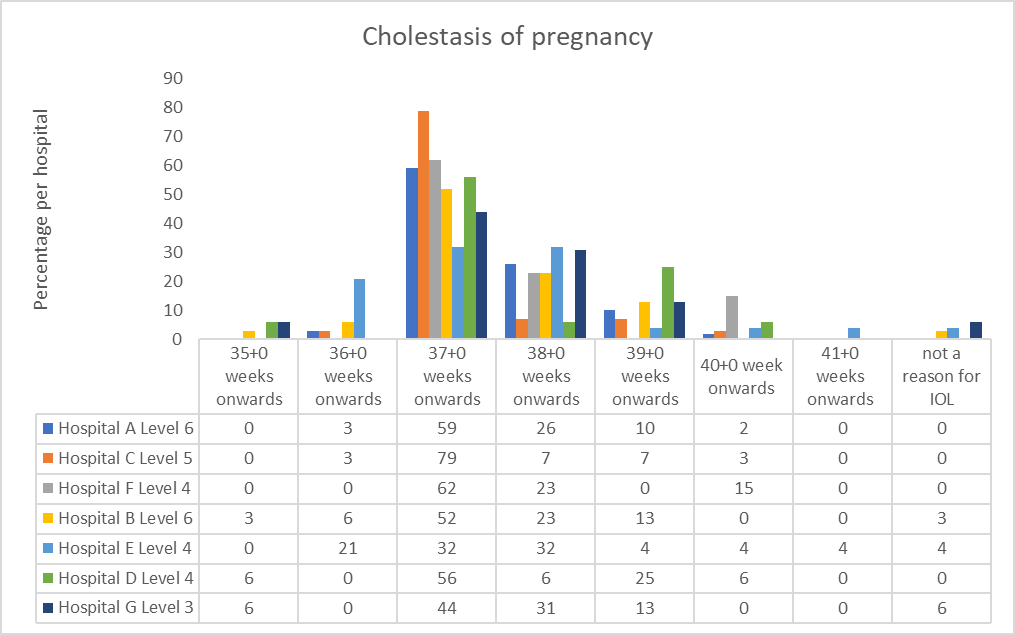
**

Maternal age of 40 and over

**
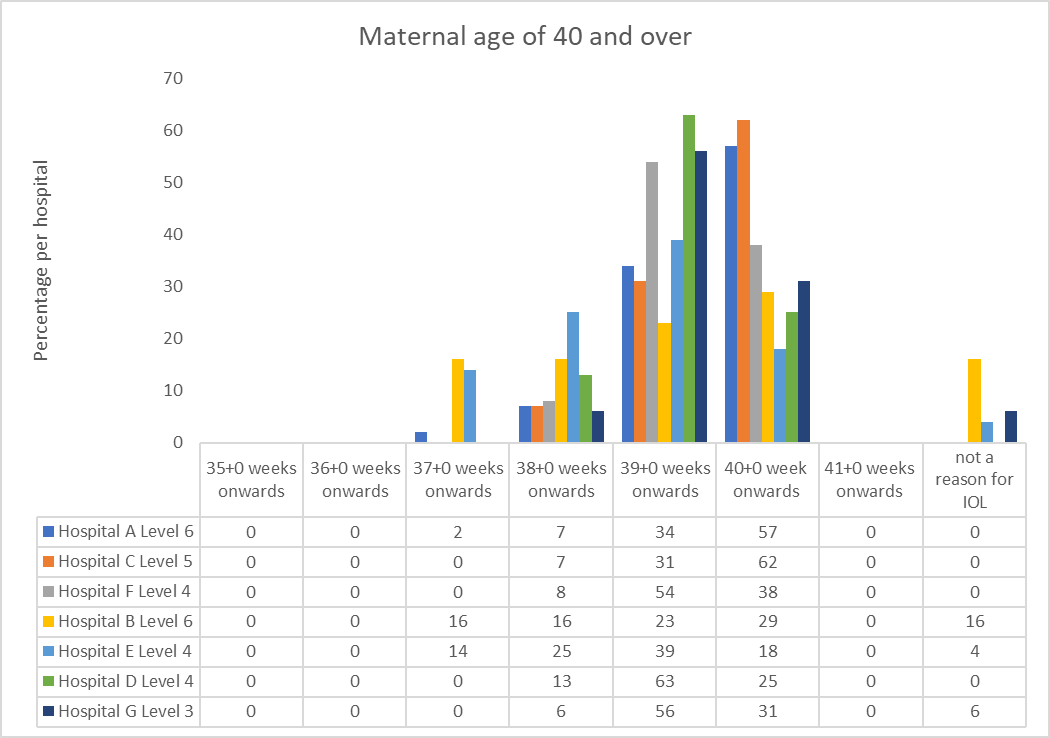
**

Substantially elevated BMI (> 40 kg/m2)

**
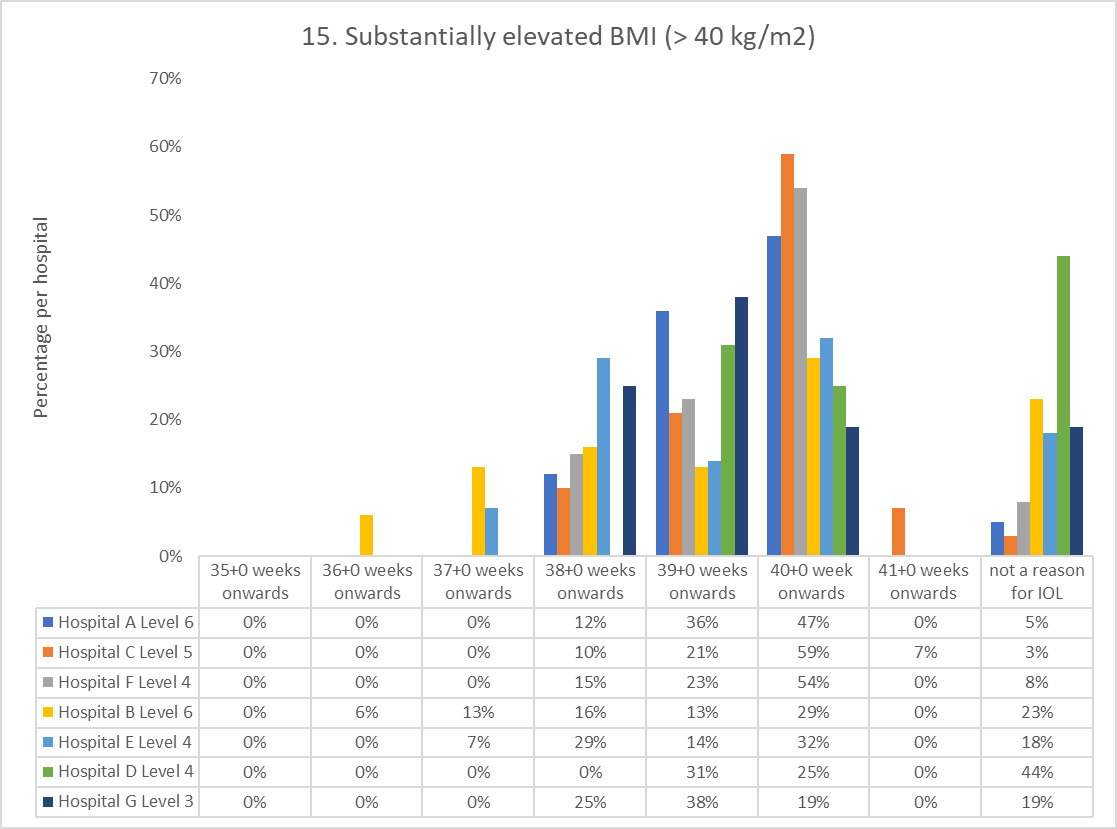
**

**Supplementary figures to Table 4**

Survey respondents’ perceptions of practices in their unit in relation to the timing of IOL for *prelabour rupture of membranes (PROM)* *women with no other complications or signs of chorioamnionitis*

A**t term** (37+0) who are Group B streptococcus positive

**
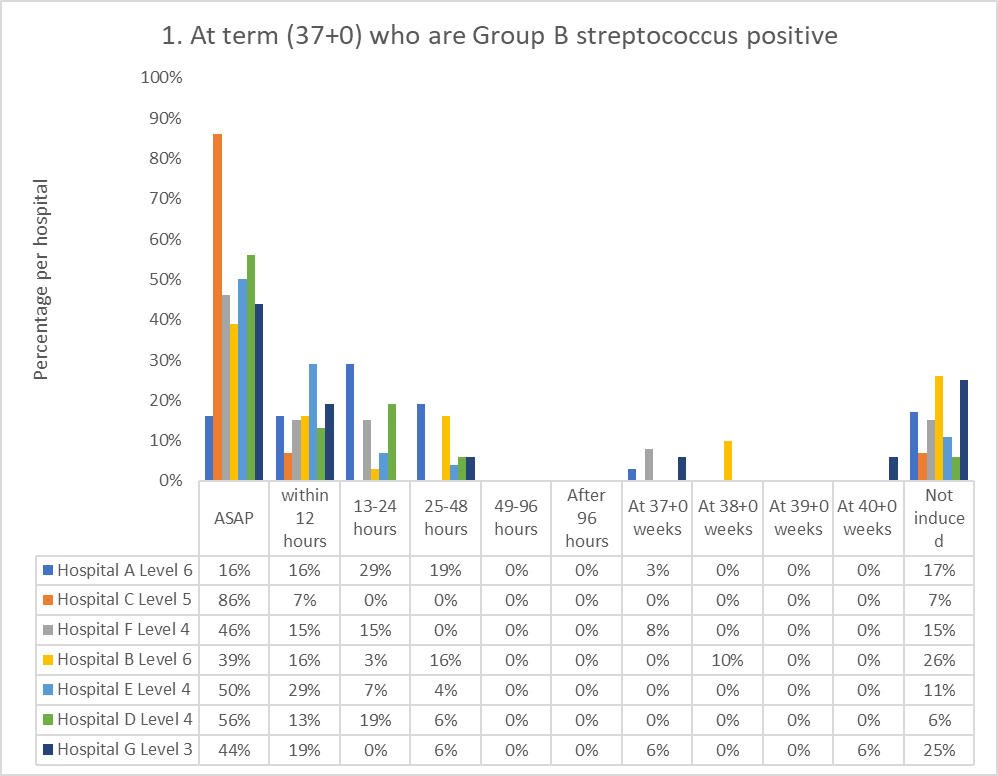
**

A**t term** (>37+0) who are Group B streptococcus negative

**
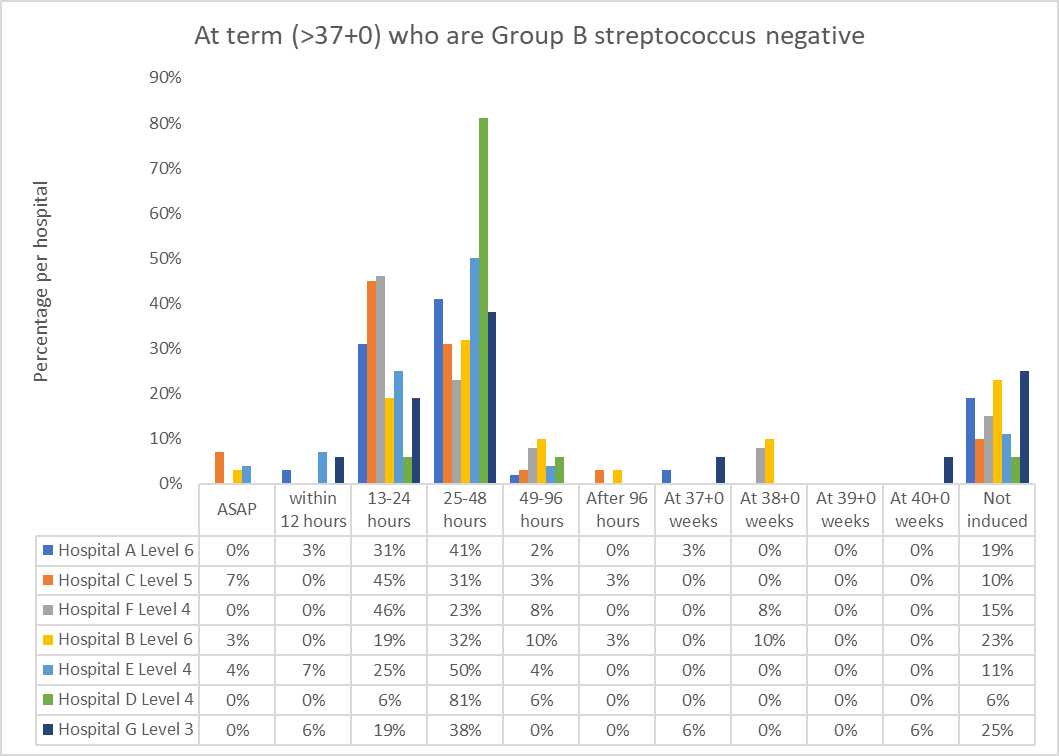
**

**Late preterm** (34+0 - 36+6 weeks) who are Group B streptococcus positive

**
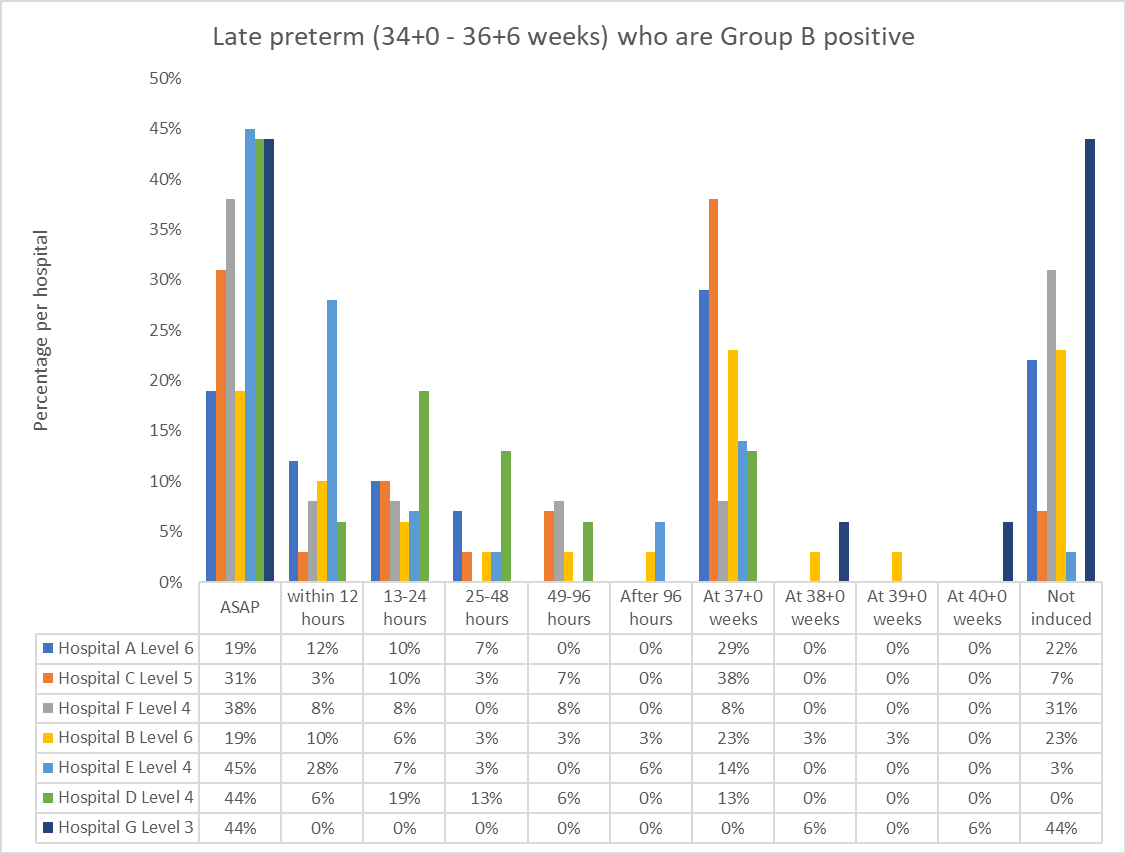
**

**Late preterm** (34+0-36+6 weeks) who are Group B streptococcus negative

**
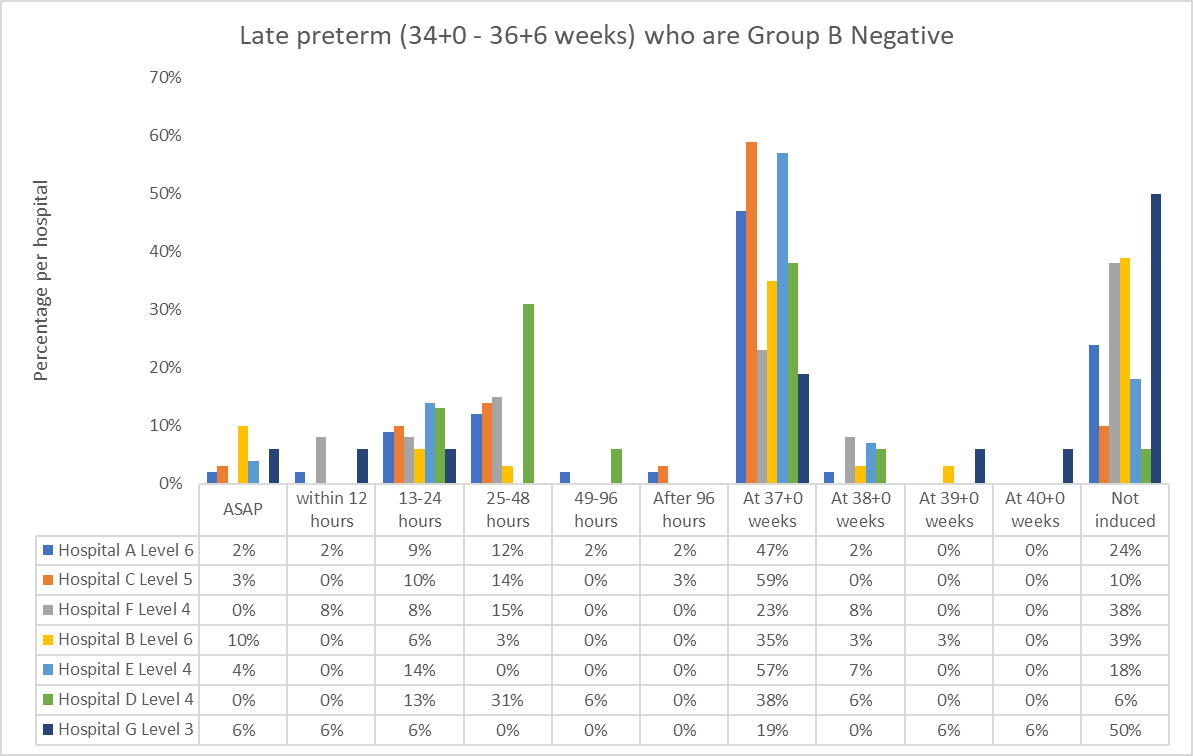
**
